# Adjunctive beneficial effect of c-di-GMP, a STING agonist, in enhancing protective efficacy of TLR4-adjuvanted tuberculosis subunit vaccine formulations

**DOI:** 10.1186/s12929-025-01144-8

**Published:** 2025-05-26

**Authors:** Kee Woong Kwon, Eunsol Choi, Hagyu Kim, Hyeong Woo Kim, Sangwon Choi, Seunghyun Lee, Sang-Jun Ha, Sung Jae Shin

**Affiliations:** 1 Department of Microbiology and Convergence of Medical Science, College of Medicine, Gyeongsang National University, Jinju, 52727 Republic of Korea; 2 Department of Microbiology, Graduate School of Medical Science, Brain Korea 21 Project, Yonsei University College of Medicine, Seoul, 03722 South Korea; 3 Department of Biochemistry, College of Life Science & Biotechnology, Yonsei University, Seoul, 03722 South Korea; 4 Brain Korea 21 (BK21) FOUR Program, Yonsei Education & Research Center for Biosystems, Yonsei University, Seoul, 03722 Republic of Korea; 5 Institute for Immunology and Immunological Disease, Yonsei University College of Medicine, Seoul, 03722 South Korea

**Keywords:** Tuberculosis, *Mycobacterium tuberculosis*, Subunit vaccine, TLR4 adjuvant, STING agonist, c-di-GMP

## Abstract

**Background:**

Effective subunit vaccine development requires selecting appropriate adjuvant formulations to trigger desired adaptive immune responses. This study explores the immunogenicity and tuberculosis (TB) vaccine potential of antigens (Ags) combined with Toll-like receptor 4 (TLR4) adjuvants and a stimulator of interferon genes (STING) agonist.

**Methods:**

In this work, we investigated the combination of Ags with TLR4 adjuvants (monophosphoryl lipid A / dimethyldioctadecylammonium bromide; MPL/DDA or glucopyranosyl lipid adjuvant-stable emulsion; GLA-SE) and a STING agonist, c-di-GMP (CDG). Mice were immunized three times by intramuscular injections at 3-week intervals. The effects of integrating Ags in these adjuvant formulations on the immune response were evaluated, focusing on the generation of Th1-biased, polyfunctional Ag-specific CD4^+^ T cells and their localization in the lung and spleen. To assess protection, immunized mice were aerogenically challenged with either conventional or ultra-low doses of *Mycobacterium tuberculosis* (Mtb) 4 weeks after the last immunization. Subsequently, bacterial load and pulmonary inflammation were assessed.

**Results:**

Integrating ESAT6 Ag in TLR4 and CDG adjuvant formulations remarkably boosted Th1-biased, polyfunctional ESAT6-specific CD4^+^ T cells in the lungs and spleen, providing durable protection against Mtb infection. The inclusion of CDG promoted mucosal localization of ESAT6-specific CD4^+^ T cells resembling resident memory phenotypes in the lung parenchyma and increased Ag-specific CD4^+^ T cells in lung vasculature. Immunization with another vaccine Ag candidate, Ag85B, in GLA-SE plus CDG similarly increased Ag85B-specific CD4^+^ T cells in the spleen and both lung compartments. Following ultra-low dose Mtb challenge, ESAT6 or Ag85B/GLA-SE/CDG immunizations significantly reduced bacterial loads compared to non-, Bacillus Calmette–Guérin (BCG)-, and ESAT6 or Ag85B/GLA-SE-immunized groups. Importantly, the inclusion of CDG decreased killer cell lectin-like receptor subfamily G member 1 (KLRG1) expression among Ag-specific CD4^+^ T cells in the lung, correlating with enhanced lung-homing evidenced by expanded lung parenchyma Ag-specific CD4^+^ T cells, including less-differentiated Th1 cells.

**Conclusions:**

This study highlights that CDG, when used in combination with TLR4 adjuvants, enhances long-term protective immunity, offering a promising strategy for subunit TB vaccine development.

**Graphical Abstract:**

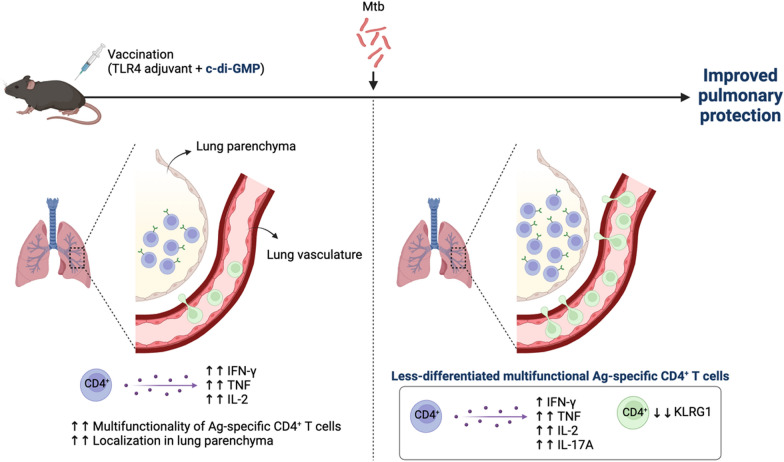

**Supplementary Information:**

The online version contains supplementary material available at 10.1186/s12929-025-01144-8.

## Introduction

*Mycobacterium tuberculosis* (Mtb) is a causative agent of tuberculosis (TB) which remains as one of the deadliest infectious diseases worldwide, displaying in higher morbidity and mortality. It causes approximately 10 million new cases of active TB and 1.3 million deaths per year in 2023 single year [1]. In addition, the effects of COVID-19 related disruptions on TB diagnosis and treatment have been catastrophic, resulting in a continued reversal of progress in controlling the TB epidemic, a trend that had been sustained for many years up to 2020. It is notable that nearly 25% of the world's population is estimated to carry a latent Mtb infection. Additionally, about 5–10% of these individuals are at an increased risk of developing an active infection during their lifetime [1–3]. This poses a substantial challenge for global TB control efforts, given the large number of individuals at risk of progressing to active TB. First introduced in 1921, Bacillus Calmette–Guérin (BCG) remains the sole clinically approved vaccine for TB. The BCG vaccine has consistently exhibited high efficacy in preventing childhood TB meningitis and miliary tuberculosis. However, its effectiveness in preventing the most prevalent and contagious form of TB, pulmonary TB, throughout all age groups has been variable, resulting in difficulties in controlling TB [4–7]. Thus, development of improved vaccine which replaces BCG for immune-compromised individuals where BCG immunization is not recommended, or aids BCG-primed effect is urgently required because prevention of TB with vaccination is the most cost-effective way.

Among the most promising strategies are protein subunit vaccines, which exhibit specificity, safety, and desirable characteristics for easy production [8–10]. These protein subunit vaccines have been reported to elicit a Th1 polarized immune response, which is primarily related with protection against Mtb infection [10–12]. Such subunit vaccine-derived immune responses have been further characterized by the contribution of the multifunctional Th1-mediated immune response co-producing IFN-γ, TNF and/or IL-2 [10, 13, 14]. Due to these features, several antigens (Ags) based subunit vaccines have currently gained the upper hand in clinical trials [8, 15–17]. Although subunit vaccines appear to possess many desirable qualities, the capability eliciting potent immune responses is weak compared to that of whole cell preparations [18]. This is because vaccination with an Ag alone is often insufficient for inducing protective immune responses. Thus, adjuvants are critical for usage with subunit vaccines in terms of increasing the qualities, duration and magnitude of adaptive immunity [19–21].

Adjuvants are basically characterized as components capable of orchestrating and/or increasing Ag-specific immune responses. They can be separated into two classes; (1) the delivery systems (e.g. liposomes, microparticles and emulsions) (2) the immune-stimulatory molecules which enhance the Ag-derived immune responses by affecting the host immune system [22, 23]. Th1 type cellular immunity is reported to be important for controlling Mtb infection [24], and thus the development of adjuvant for targeting TB aims to elicit robust Th1 immune response. Importantly, only few adjuvants are approved for use in human, and the most of them are poor inducers of Th1 polarized cellular immunity [11, 22]. Therefore, the investigation for new adjuvants is regarded as high priority for vaccinologists.

As one of approaches for enhancing Th1 responses, the incorporation of molecules that are able to interact with the pattern recognition receptors (PRRs) of the innate immune system, especially Toll-like receptors (TLRs) and nucleotide-binding oligomerization domain (NOD)-like receptors (NLRs), to recognize pathogen-associated molecular patterns (PAMPs) [25, 26]. Some advanced subunit vaccines against TB in clinical trials adopted immune-stimulatory molecules as adjuvant that are TLR agonist. For example, M72 vaccine use the liposomal formulated AS01E adjuvant which is developed by GlaxoSmithKline (GSK). This AS01E adjuvant consists of the detergent QS-21 and monophosphoryl lipid A (MPL), and MPL has been reported to function as TLR4 agonist [27–29]. As another example, the ID93 vaccine, created by the Infectious Disease Research Institute (IDRI), includes glucopyranosyl lipid adjuvant-stable emulsion (GLA-SE), a well-defined TLR4 agonist, as an adjuvant [30–32]. The Statens Serum Institute (SSI)-derived IC31 adjuvant is based on a TLR9 agonist combined with a cationic peptide [28]. In addition, the CAF01 adjuvant, targeting other PRRs such as C-type lectin receptors, and polysaccharide adjuvants beyond the signaling pathway, such as Advax, have shown novel adjuvant potential against Mtb infections [33, 34].

Although cell membrane associated TLR4 and endosomal TLR9 detect extracellular and vacuolar pathogens, the productive growth of several pathogens such as intracellular bacteria happen in the cytosol [35]. This necessitates the requirement of using agonist that activates cytosolic PRRs. Especially, PRRs sensing nucleic acid have been recently identified [36]. Stimulator of interferon (IFN) genes (STING) has been defined as an adaptor protein in response to cytosolic DNA by mediating intracellular signaling pathways followed by the activation of nuclear factor kappa B (NF-B), interferon regulatory factor 3 (IRF3) and Tank-binding kinase-1 (TBK1) respectively, resulting in autophagy activation or production of IFN-β [37]. Mtb has been also reported to activate cytosolic PRRs through phagosomal disruption by the mycobacterial protein secretion system ESX-1-derived virulence factors such as ESAT-6 and CFP-10 [38, 39]. Then, mycobacterial DNA can access into cytosol, resulting in cytokine production indirectly through STING-TBK1-IRF3 axis [38]. More recently, cyclic-di-GMP-AMP synthase (cGAS), upstream of STING, has been identified as the main DNA sensor and mediates catalytic reactions by synthesizing cyclic-di-GMP-AMP (cGAMP) from GTP and ATP subsequent to binding and activation of STING [36]. Through this discovery, cGAS has been demonstrated to function as direct innate immune DNA sensor for Mtb [37]. Moreover, STING, the downstream of cGAS, is able to bind cyclic dinucleotides and bacterial second messenger such as cyclic di-AMP (c-di-AMP) and cyclic di-GMP (CDG) [40]. Notably, these STING agonists have been shown marked potential use as novel vaccine adjuvants [35, 41, 42].

In this study, we explored the potential of c-di-GMP (CDG), a STING pathway agonist, as a novel adjuvant with vaccine Ag. We assessed its efficacy when co-formulated with established TLR4 adjuvants and evaluated its capacity to enhance the protective immunity of TLR4-adjuvanted TB subunit vaccines against infections with Mtb clinical isolates in mice.

## Materials and methods

### Mice

Specific pathogen-free (SPF) female C57BL/6 J mice at 6–7 weeks of age were purchased from Japan SLC, Inc. (Shizuoka, Japan) and maintained under barrier conditions in the ABSL-3 facility at the Avison Biomedical Research Center of the Yonsei college of Medicine. All mice were housed in a constant temperature/humidity environment (24 ± 1 °C, 50 ± 5%) under light-controlled conditions (12 h light–dark cycle; 7 am on and 7 pm off) and fed a sterile commercial mouse diet with ad libitum access to water.

### Preparation of Mycobacterial strains

Mtb K strain was obtained from the strain collections at the Korean Institute of Tuberculosis (KIT, Osong, Chungchungbuk-do, Korea), and the Mtb HN878 strain was obtained from the strain collections of the International Tuberculosis Research Center (ITRC, Changwon, Gyeongsangnam-do, Korea). BCG (Pasteur 1173P2) was kindly provided by Dr. Brosch at the Pasteur Institute (Paris, France). All mycobacterial strain used in this study was cultured and prepared as described previously [3].

### Expression and purification of recombinant proteins

Recombinant ESAT6 and Ag85B were prepared as described previously [3, 43]. Briefly, the *esat6* and *ag85b* were amplified from Mtb H37Rv ATCC27294 genomic DNA using following primer sets: *esat6* forward, 5’-AAGCTTATGACAGAGCAGCAGTGGAAT-3’ (*HindIII*), and reverse, 5’-CTCGAGTGCGAACATCCCAGTGACGTT-3’ (*XhoI*); *ag85b* forward, 5’-CATATGACAGACGTGAGCCGAAAGATT-3’ (*NdeI*), and reverse, 5’- AAGCTTGCCGGCGCCTAACGAACTCTG-3’ (*HindIII*). The DNA fragments of ESAT6 and Ag85B were respectively cloned into the restriction sites (ESAT6; *HindIII* and *Xho I*, Ag85B; *NdeI* and *HindIII*) of the plasmid pET22b ( +) vector (Novagen, Madison, WI, USA), and the c-terminal His-tag present in the plasmid was included. The product was transformed into *Escherichia coli* BL21, which was induced to express proteins by isopropyl β-D-1-thiogalactopyranoside (IPTG). IPTG was added at a final concentration of 1 mM. The overexpressed ESAT6 and Ag85B proteins were prepared with cell disruption by sonication and purified using nickel-nitrilotriacetic acid (Ni–NTA) resin. To remove endotoxin contamination from the purified protein, the dialyzed recombinant protein was incubated with polymyxin B-agarose (Sigma, St. Louis, MO, USA) for 6 h at 4 °C. Purified endotoxin-free proteins were filter sterilized and frozen at − 70 °C. The protein concentration was estimated with the BCA kit (Pierce, Rockford, IL, USA). Residual LPS in the ESAT6 and Ag85B preparations was determined using the Limulus amebocyte lysate (LAL) test (Lonza, Basel, Switzerland), according to the manufacturer’s instructions. The purity of ESAT6 and Ag85B was evaluated by Coomassie blue staining and Western blot using an anti-histidine antibody (Fig. S1).

### Immunization and Mtb infection in mice

Mice were immunized three times by intramuscular injections with three weeks intervals. The subunit vaccine was formulated with two ways with different adjuvants: (1) 1 μg of ESAT6, 5 μg of c-di-GMP and 50 μg of dimethyldioctadecylammonium (DDA) containing 5 μg of monophosphoryl lipid-A (MPL); (2) 1 μg of ESAT6 or Ag85B, 5 μg of c-di-GMP and 5 μg of GLA-SE. Adjuvant control groups were immunized with MPL/DDA and GLA-SE respectively. MPL and DDA were purchased from Sigma-Aldrich (St. Louis, MO, USA). GLA-SE was purchased from IDRI (Seattle, WA, USA). For BCG immunization, 2 × 10^5^ CFU of BCG Pasteur 1173P2 were given via subcutaneous route. Four weeks after the last immunization, immunized mice were aerogenically infected with Mtb K or HN878 strains as previously described [3]. Briefly, the calibrated inhalation chamber of airborne infection apparatus (Glas-Col, Terre Haute, IN, USA) was used to deliver an approximately 200 (conventional dose; Mtb K, and HN878 strains) or 10 (ultra-low dose; Mtb K strain) viable bacteria. At the indicated time point, mice from each group were euthanized in a euthanasia chamber following the gradual carbon dioxide (CO_2_) filling method from a compressed CO_2_ gas cylinder, followed by KFDA guidelines for subsequent analysis.

### Antibody titers in serum

96-well plates were coated with 1 μg/ml of ESAT-6, and Ag-specific IgG, IgG1 and IgG2c responses in serum were measured as previously described [44].

### In vivo intravascular labeling

For intravenous staining, mice were injected intravenously with FITC-conjugated anti-CD45.2 (104; BD Biosciences). Four microgram of antibody was used per mouse in a total volume of 200 μl. Three minutes after antibody injection, mice were euthanized as described above, and lung single cell suspensions were prepared as described below. Peripheral blood mononuclear cells (PBMCs) were isolated from blood by density gradient centrifugation using Histopaque-1077 (Sigma-Aldrich) according to the manufacturer’s protocol. Bronchoalveolar lavage fluid (BALF) was first acquired using 1 ml of cold PBS through the murine trachea. The aspiration was repeated twice until no further fluid was collected. BALF was centrifuged, and the pellets were used for analysis. These harvested PBMCs and BALFs were used as positive and negative controls, respectively (Figs. S2A and S2B). In addition, for immunofluorescence staining, mice were co-injected intravenously with Alexa594-conjugated anti-CD45.2 and Alexa647-conjugated anti-CD31 (Biolegend), and the lung tissue cryosections (5 mm) were cut from 4% paraformaldehyde-fixed, cryopreserved with 30% sucrose, optimal cutting temperature (OCT) compound (Tissue Tek, Torrance, USA)-embedded blocks. Slides were mounted with UltraCruz Aqueous Mounting Medium with DAPI (Santa Cruz, Dallas, Texas, USA). Images were captured with a Zeiss LSM 700 confocal laser microscope (Fig. S2C).

### Tetramer, surface and intracellular cytokine staining

Tetramers (ESAT-6_4–17_:I-A(b) and Ag85B_280-294_:I-A(b)) conjugated to PE and corresponding negative controls (hCLIP:I-A(b)) were provided by the NIH tetramer facility (Atlanta, USA). Single cell suspensions of the lungs and spleens were prepared as previously described [3]. Single cells suspensions (1.0 × 10^6^ cells) from the lungs and spleen of each group were stained with MHC-II tetramers (ESAT6 and Ag85B: 1:100) for 30 min at 37 °C. Then single cell suspensions were blocked with anti-CD16/32 (BD Bioscience, San Diego, CA) for 15 min at 4 °C. For phenotypical analysis, single cell suspensions were stained with the following antibodies for 20 min at 4 °C; Thermo Fisher Scientific (Waltham, MA, USA): Live/Dead Fixable Viability Dye eFluorTM 780; BD Biosciences: anti-CD90.2 (53–2.1)-BUV805, anti-CD4 (RM4-5)-BV650, anti-CD4 (RM4-5)-PerCP-Cy5.5, anti-CD8a (53–6.7)-BV786, anti-CD62L (MEL-14)-APC-R700, anti-CD44 (IM7)-BV480; Biolegend: anti-CD90.2 (53–2.1)-BV605, anti-CD44 (IM7)-PE-Cy7, anti-CD44 (IM7)-BV421, anti-CD62L (MEL-14)-Alexa700, anti-CD69 (H1.2F3)-PE/Dazzle 594, anti-KLRG1 (2F1/KLRG1)-BV605. For intracellular staining, cells were fixed, permeabilized, and stained intracellularly with following antibodies; Biolegend: anti-IFN-γ (XMG1.2)-BV421, anti-IL-2 (JES6-5H4)-PE/Dazzle 594, anti-TNF (MP6-XT22)-APC. To detect intracellular cytokines in T cells from the lungs and spleen, single cell suspensions (1.0 × 10^6^ cells) were stimulated following ESAT6 at 37 °C for 9 h in the presence of both GolgiPlug and GolgiStop (BD Biosciences). Then, cells were analyzed using the FACSymphony A3 (BD Biosciences) and CytoFLEX S flow cytometer (Beckman Coulter, Indianapolis, IN, USA), and subsequent analysis was performed using FlowJo version 10 software (TreeStar, Ashland, OR, USA).

### Colony forming units (CFU) and histopathology

Both the lungs and spleen from the infected mice were homogenized as previously described [44]. Then serial dilutions were plated on 7H11 plates (Becton Dickinson, Franklin Lakes, NJ, USA) supplemented with amphotericin B and 10% OADC (Difco Laboratories). CFUs were enumerated 4 weeks incubation at 37 °C. For histopathological analysis, the middle cross-section from the entire superior lobes of the right lung were stained with hematoxylin and eosin (H&E) and assessed for the severity of inflammation. The severity of lung inflammation was determined using ImageJ (National Institutes of Health, USA) and Adobe Photoshop (Adobe, San Jose, California) as previously described [3, 45]. Briefly, we isolated the complete lung lesion image in Adobe Photoshop and saved it as a separate file, along with a black-filled image of identical size. These files were then opened in ImageJ, where the 'Split Channels' function processed the green channel image to measure the area of green positivity, which appeared purple-blue due to H&E staining. Similarly, the black-filled image was analyzed via the green channel to calculate the total lung lesion area. The 'inflamed area (%)', representing the proportion of inflamed tissue relative to the total lung lesion, was determined by dividing the green positivity area of the lesion image by that of the black-filled image.

### Assessment of cytokine production

The levels of secreted TNF, IL-4, IL-17A, IL-2, IFN-γ (Thermo Fisher), IL-10 (BD Bioscience) in the cell culture supernatant were detected with a commercial enzyme-linked immunosorbent assay (ELISA) kit according to the manufacturer’s instructions. Single cell prepared from the lungs of Mtb-infected mice were stimulated with ESAT-6 or Ag85B (1 μg/ml) for 12 h at 37 °C.

### Statistical analysis

Data for all experiments are presented as the mean ± SD. For immunological analysis, the levels of significance for comparison between samples were determined by Tukey’s multiple comparison or unpaired *t*-test. For CFU and histopathology analysis, the Mann–Whitney rank test was used when comparing the differences between two different groups. For statistical analysis, GraphPad Prism version 8.00 for Windows was used (GraphPad Software, La Jolla California USA, www.graphpad.com). Differences having *p < 0.05, **p < 0.01, ***p < 0.001, or ****p < 0.0001 were considered statistically significant. The statistical details, including the statistical test, exact value of n, precision measure and statistical significance threshold, are reported in the figures and figure legends.

## Results

### Enhanced immunogenicity by combining CDG with a TLR adjuvant, MPL

**Fig. 1:**
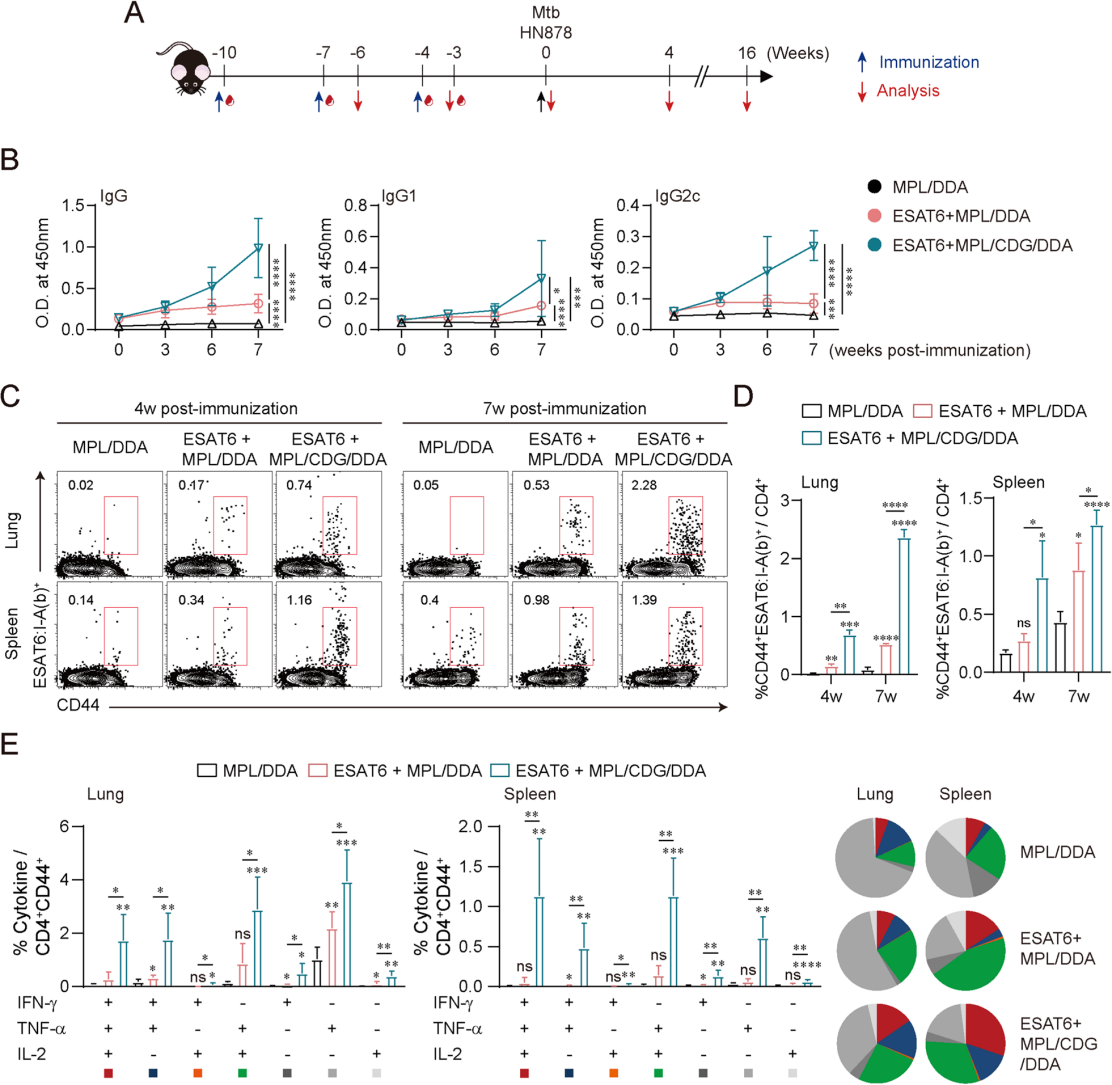
Analysis of ESAT6-specificimmunogenicities in ESAT6 + MPL/c-di-GMP (CDG)/DDA-immunized mice. **A** Experimental scheme of the in vivo experiment. **B** Individual mouse sera (*n* = 4) were isolated and diluted 1000-fold at the indicated time points after the first immunization. ESAT6-specific IgG, IgG1, and IgG2c levels were then measured with an ELISA. **C** Ag-specific CD4^+^CD44^+^ T cells of lungs and spleen from each group (*n* = 3) were evaluated with the use of ESAT6-tetramer. The numbers in plots indicate the frequency of ESAT6-specific CD4^+^CD44^+^ T cells. **D** The frequency of ESAT6-specific CD4^+^CD44^+^ T cells in lungs and spleen from each group were summarized in bar graphs. **E** ESAT6-stimulated lung cells and splenocytes from each subset of mice (*n* = 4) were assessed based on the frequency of CD4^+^CD44^+^ T cells with different patterns of cytokine production and presented as bar graph (left panel). The pie charts (right) summarized the fraction of triple, double, and single cytokine-positive CD4^+^ T cell in each group. Graphs show the means ± SD. The data are representative of a single experiment. n.s. not significant, *p < 0.05, **p < 0.01, ***p < 0.001, and ****p < 0.0001 compared to MPL/DDA only immunized mice. *p < 0.05, **p < 0.01, ***p < 0.001, and ****p < 0.0001 between ESAT6 + MPL/DDA- and ESAT6 + MPL/CDG/DDA-immunized mice

Firstly, we investigated whether the STING agonist, CDG, possesses adjuvant potential when combining with Mtb antigen in vivo. Thus, the adjuvant effect of CDG was compared with MPL, a TLR4 agonist, by formulating it with the ESAT6 antigen (Fig. S3A). At 4 weeks after the final immunization, a similar enhancement of ESAT6-specific IgG, IgG1, and IgG2c titers was observed in ESAT6 + CDG/DDA-immunized mice compared to those from ESAT6 + MPL/DDA immunization (Fig. S3B). Moreover, ESAT6 + CDG/DDA immunization significantly conferred long-term protection, comparable to the protection mediated by ESAT6 + MPL/DDA immunization, against Mtb clinical isolate HN878 infection, as evidenced by reduced pulmonary inflammation and bacterial loads (Figs. S3C and S3D). Based on the adjuvanticity derived from CDG, we next evaluated the combined effect of CDG on the MPL adjuvant (Fig. 1A). Upon assessing humoral responses at 3 weeks after the first immunization, a remarkable induction of ESAT6-specific IgG titers was observed in ESAT6 + MPL/CDG/DDA-immunized mice. Notably, the Ag-specific IgG2c response was more pronounced in ESAT6 + MPL/CDG/DDA-immunized mice than the IgG1 response (Fig. 1B). Using ESAT-6_4–17_:I-A(b) tetramer staining, a rapid and robust generation of ESAT6-specific CD4^+^ T cells was observed in both the lungs and spleen of mice immunized with ESAT6 + MPL/CDG/DDA (Fig. 1C and D). Given the protective contribution of polyfunctional T cells in eradicating intracellular pathogens [3], Ag-specific polyfunctional T cells were further analyzed among CD4^+^ T cells collected from the lungs and spleen using multicolor flow cytometry (following the gating strategies outlined in Fig. S4). When stimulated with ESAT6, mice immunized with ESAT6 + MPL/CDG/DDA exhibited remarkably enhanced levels of CD4^+^ T cells secreting IFN-γ, TNF, and/or IL-2 in both the lungs and spleen compared to those from ESAT6 + MPL/DDA immunization (Fig. 1E). Additionally, immunization with ESAT6 adjuvanted with MPL/DDA plus CDG elicited Ag-specific Th1-biased responses by inducing significantly higher Th1-related IFN-γ production compared to Th2-related IL-5 production (Fig. S5). In summary, the inclusion of CDG accelerates the abundant generation of Ag-specific CD4^+^ T cells along with elevated Th1-biased polyfunctional properties in both the lungs and spleen.

### Improved protection conferred by CDG addition against Mtb clinical isolate HN878 infection

**Fig. 2:**
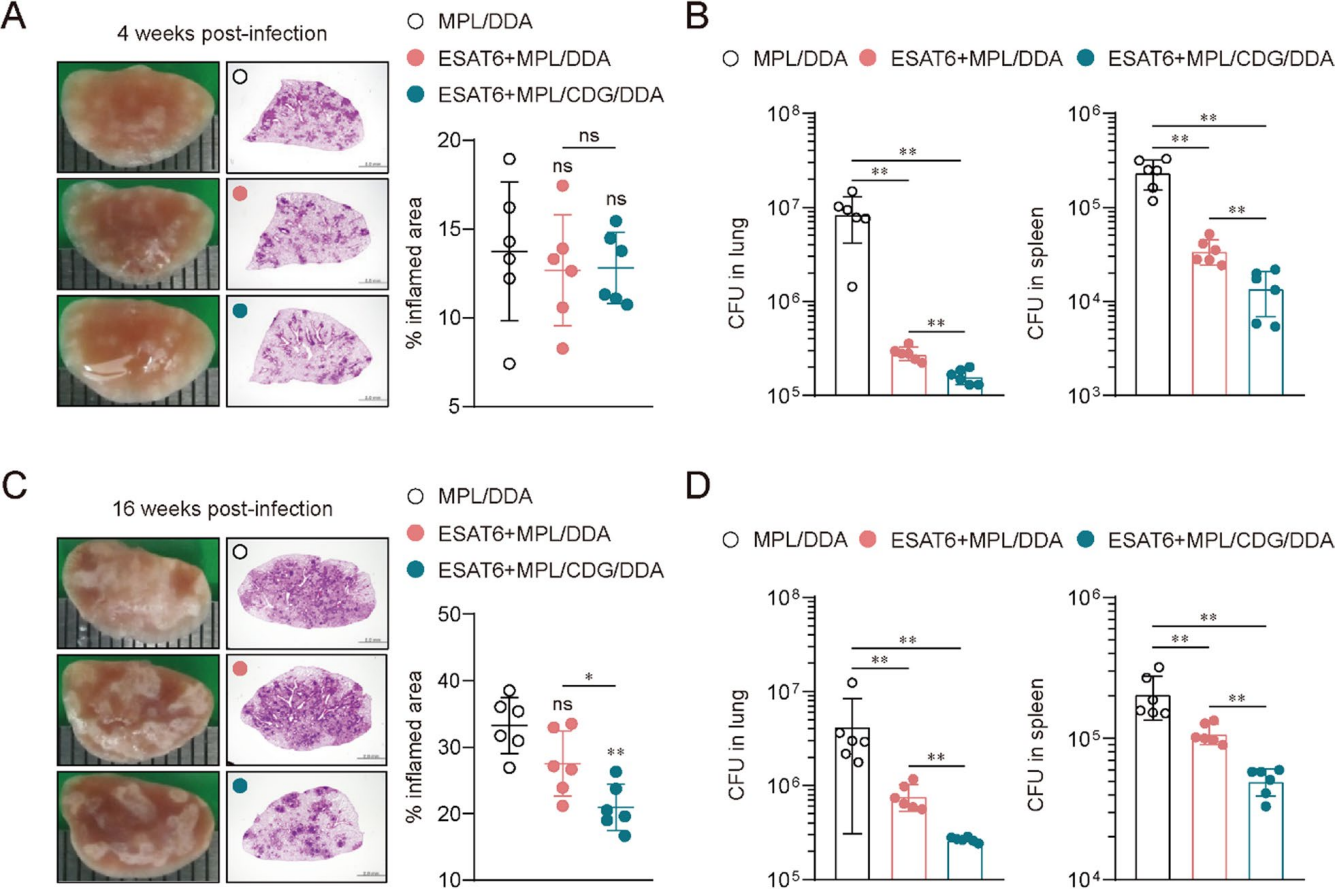
Protective efficacy of ESAT6 subunit vaccine formulated in MPL and c-di-GMP (CDG) against Mtb HN878 strain. **A** Gross pathology and H&E staining of lungs of each group (*n* = 6; 10X: scale bar = 2.0 mm) at 4 weeks post Mtb infection. The experimental results indicated the percentages of inflamed area and are described by dot plots. **B** Mtb CFU in both lungs and spleen of each group at 4 weeks post infection were analyzed by enumerating the viable bacteria. **C** Gross pathology and H&E staining of lungs of each group (*n* = 6; 10X: scale bar = 2.0 mm) at 16 weeks post Mtb infection. The experimental results indicated the percentages of inflamed area and are described by dot plots. **D** Mtb CFU in both lungs and spleen of each group at 16 weeks post infection were analyzed by enumerating the viable bacteria. Graph shows mean ± SD. The data are representative of a single experiment. Mann–Whitney rank tests were used to compare groups. n.s. not significant, *p < 0.05 and **p < 0.01

Based on the increased immunogenicity conferred by CDG addition to MPL, we then evaluated the protective efficacy of ESAT6 + MPL/CDG/DDA immunization against a highly virulent Mtb Beijing clinical isolate HN878 challenge (Fig. 1). Four weeks after Mtb HN878 infection, histopathological analysis and bacterial burden were examined in the MPL/DDA-, ESAT6 + MPL/DDA-, ESAT6 + MPL/CDG/DDA-immunized groups. The bacterial burden of the lung and spleen was significantly lower in the ESAT6 + MPL/CDG/DDA-immunized group than the ESAT6 + MPL/DDA-immunized group at 4 weeks post-challenge, although lung inflammation displayed similar level compared to other groups (Fig. 2A and B). More importantly, CDG immunized mice evidently conferred long-lasting protection at 16 weeks post-infection given that lung inflammation and CFUs were significantly reduced compared to both ESAT6 + MPL/DDA and MPL/DDA immunized mice (Fig. 2C and D). These results suggest that CDG addition potentially offers improved protective efficacy by reducing bacterial burden and inflammation during chronic infections with highly virulent Mtb.

### Augmented immunological effect conferred by CDG on GLA-SE, a well-defined TLR4 adjuvant

**Fig. 3:**
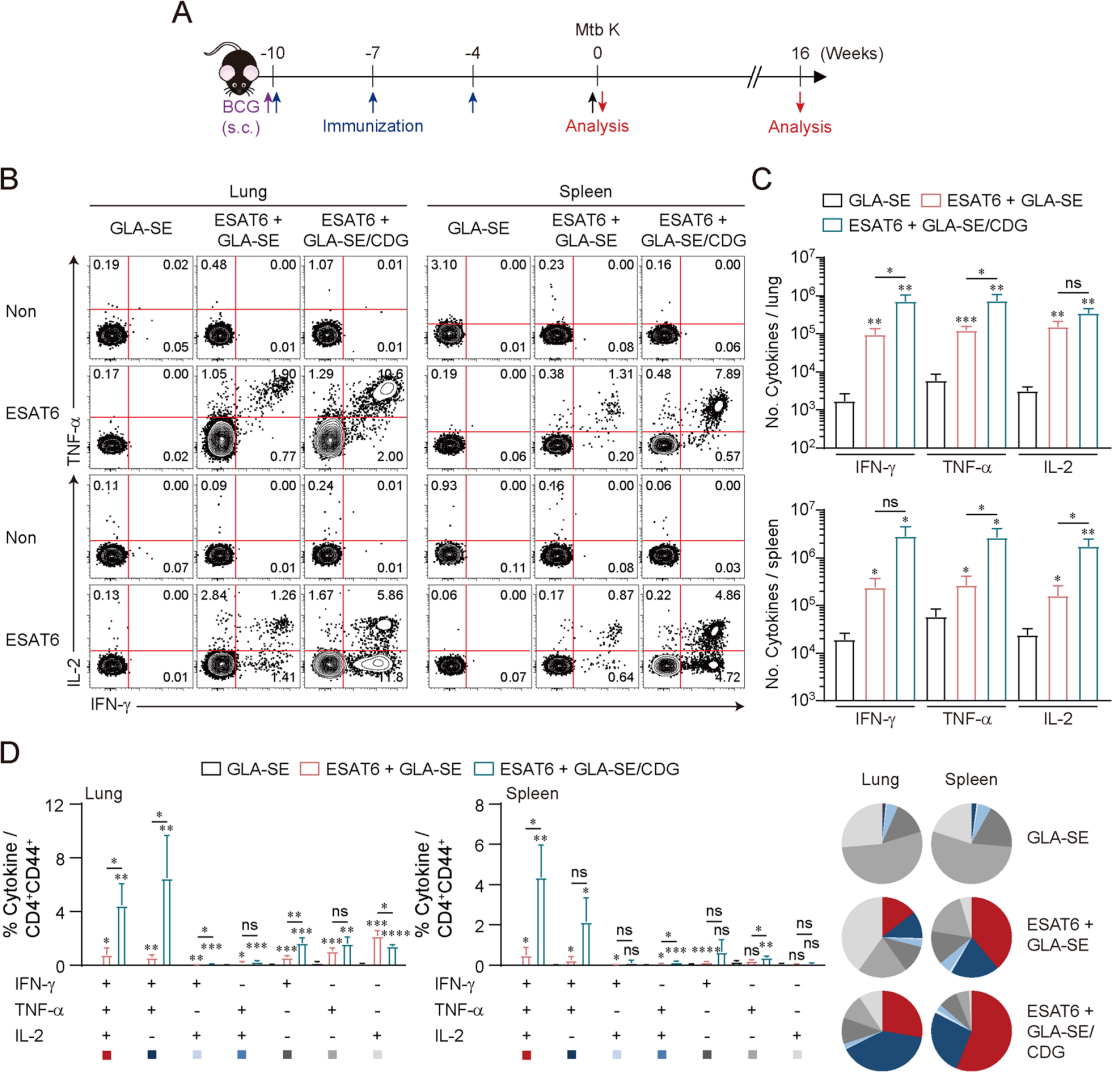
Analysis of CD4^+^ T cell responses induced by ESAT6 + GLA-SE in the presence or absence of c-di-GMP (CDG) immunization in the lungs and spleen of immunized mice. **A** Experimental scheme of the in vivo experiment. **B** Representative plots of IFN-γ^+^, TNF^+^, and IL-2^+^ in CD4^+^ T cells. The numbers in plots indicate the IFN-γ^+^TNF^+^, IFN-γ^+^TNF^−^, and IFN-γ^−^TNF^+^ / IFN-γ^+^IL-2^+^, IFN-γ^+^IL-2^−^, and IFN-γ^−^IL-2^+^. **C** The number of IFN-γ^+^, TNF^+^, or IL-2^+^ CD4^+^ T cells in lungs and spleens from each group (*n* = 3, 4) were summarized in graph. **D** ESAT6-stimulated lung cells and splenocytes from each subset of mice (*n* = 3, 4) were assessed based on the frequency of CD4^+^CD44^+^ T cells with different patterns of cytokine production and presented as bar graph (left panel). The pie charts (right) summarized the fraction of triple, double, and single cytokine-positive CD4^+^ T cell in each group. Graphs show the means ± SD. The data are representative of a single experiment. n.s. not significant, *p < 0.05, **p < 0.01, ***p < 0.001, and ****p < 0.0001 compared to GLA-SE only immunized mice. n.s. not significant, *p < 0.05, and **p < 0.01 between ESAT6 + GLA-SE- and ESAT6 + GLA-SE/CDG-immunized mice

Next, to confirm whether CDG-derived immunological boosting effect was applied to another synthetic TLR4 agonist, GLA-SE, which has been tested in a phase 2a vaccine trial [31, 32], we evaluated the adjuvanticity of GLA-SE plus CDG in a similar mouse experimental setting to Fig. 1 with another Mtb Beijing clinical isolate K from a TB outbreak in high schools in South Korea [46] (Fig. 3A). To elucidate the Ag-specific CD4^+^ T cells producing IFN-γ, TNF, and IL-2, lymphocytes from both the lungs and spleen of immunized mice were stimulated with ESAT6 followed by intracellular cytokine staining. When lymphocytes were ex vivo stimulated with ESAT6, the number of ESAT6-specific IFN-γ^+^- and TNF^+^-CD4^+^ T cells in the lungs and the number of ESAT6-specific TNF^+^- and IL-2^+^-CD4^+^ T cells in the spleen were significantly increased compared to those of the ESAT6 + GLA-SE immunized group (Fig. 3B and C). As observed in ESAT6 + MPL/CDG/DDA immunization, the inclusion of CDG in GLA-SE elicited ESAT6-specific Th1-biased CD4^+^ T cell responses (Figs. S6A and S6B). Next, we validated the induction of Ag-specific polyfunctional T cell responses. When stimulated with ESAT6, the mice immunized with GLA-SE plus CDG exhibited enhanced marked polyfunctionality of Ag-specific CD4^+^ T cells, characterized by the co-production of IFN-γ, TNF, and/or IL-2 in both the lungs and spleen (Fig. 3D), suggesting that CDG enforced GLA-SE-derived Ag-specific T cell responses.

### Durable protective efficacy achievement resulted from ESAT6 + /GLA-SE/CDG immunization

**Fig. 4:**
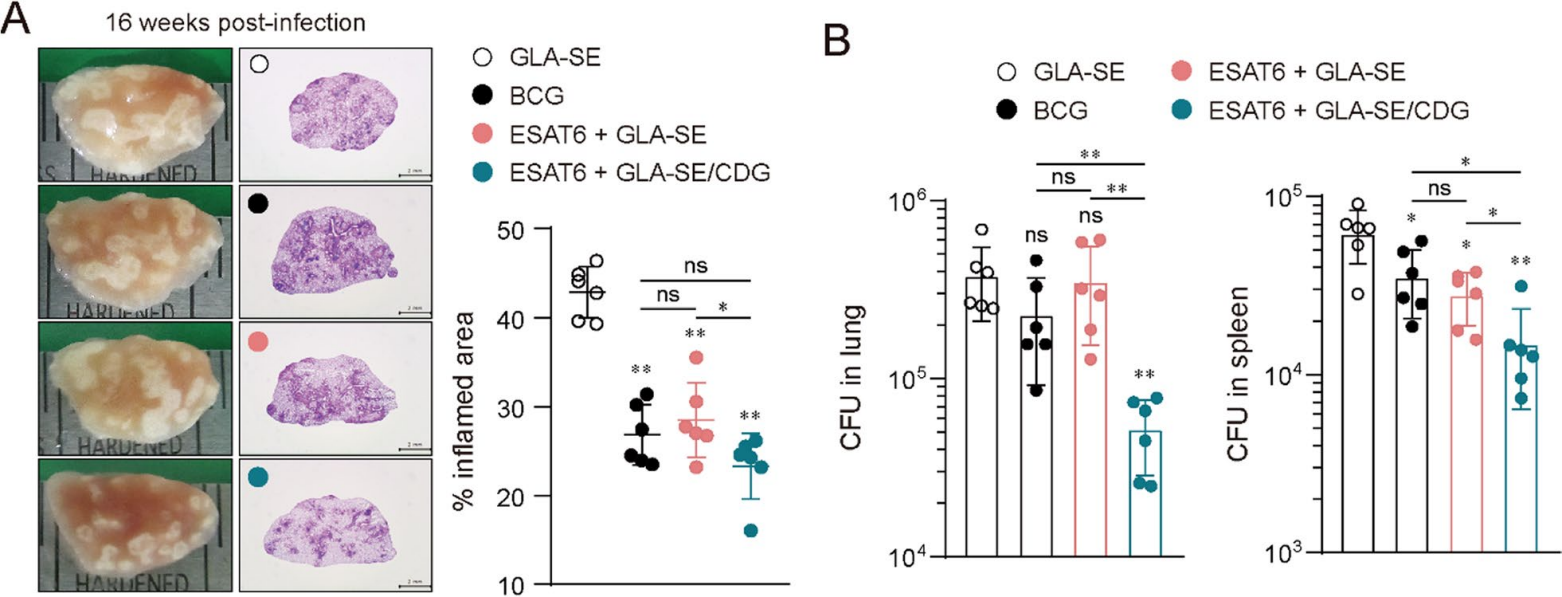
Evaluation of vaccine efficacy in mice immunized with ESAT6 + GLA-SE/c-di-GMP (CDG) against Mtb strain K infection. **A** Gross pathology and H&E staining of lungs of each group (*n* = 6; 10X: scale bar = 2.0 mm) at 16 weeks post Mtb infection. The experimental results indicated the percentages of inflamed area and are described by dot plots. **B** Mtb CFU in both lungs and spleen of each group at 16 weeks post infection were analyzed by enumerating the viable bacteria. Graph shows mean ± SD. The data are representative of a single experiment. Mann–Whitney rank tests were used to compare groups. n.s. not significant, *p < 0.05 and **p < 0.01

In line with the observations that the inclusion of CDG in MPL/DDA mediated long-term protection against Mtb HN878 infection (Fig. 2), we also confirmed that ESAT6 + GLA-SE/CDG immunization significantly reduced lung inflammation and bacterial loads compared to ESAT6 + GLA-SE immunization at 16 weeks post-Mtb K infection. Notably, ESAT6 + GLA-SE/CDG immunization significantly ameliorated pulmonary inflammation compared to the ESAT6 + GLA-SE-immunized group, achieving a level similar to that of the BCG-immunized group. Moreover, ESAT6 + GLA-SE/CDG immunization displayed superior protection, evidenced by reduced bacterial loads compared to the BCG-immunized group (Fig. 4A and B). These results indicate that CDG possesses cooperative adjuvant effects with both well-established TLR4 adjuvants, MPL/DDA and GLA-SE, against Mtb Beijing clinical isolate infections.

### The increase in Ag-specific CD4^+^ T cells in the lung compartment and spleen resulted from vaccination with the TB subunit vaccine containing common antigen Ag85B or ESAT6, adjuvanted with GLA-SE/CDG

**Fig. 5:**
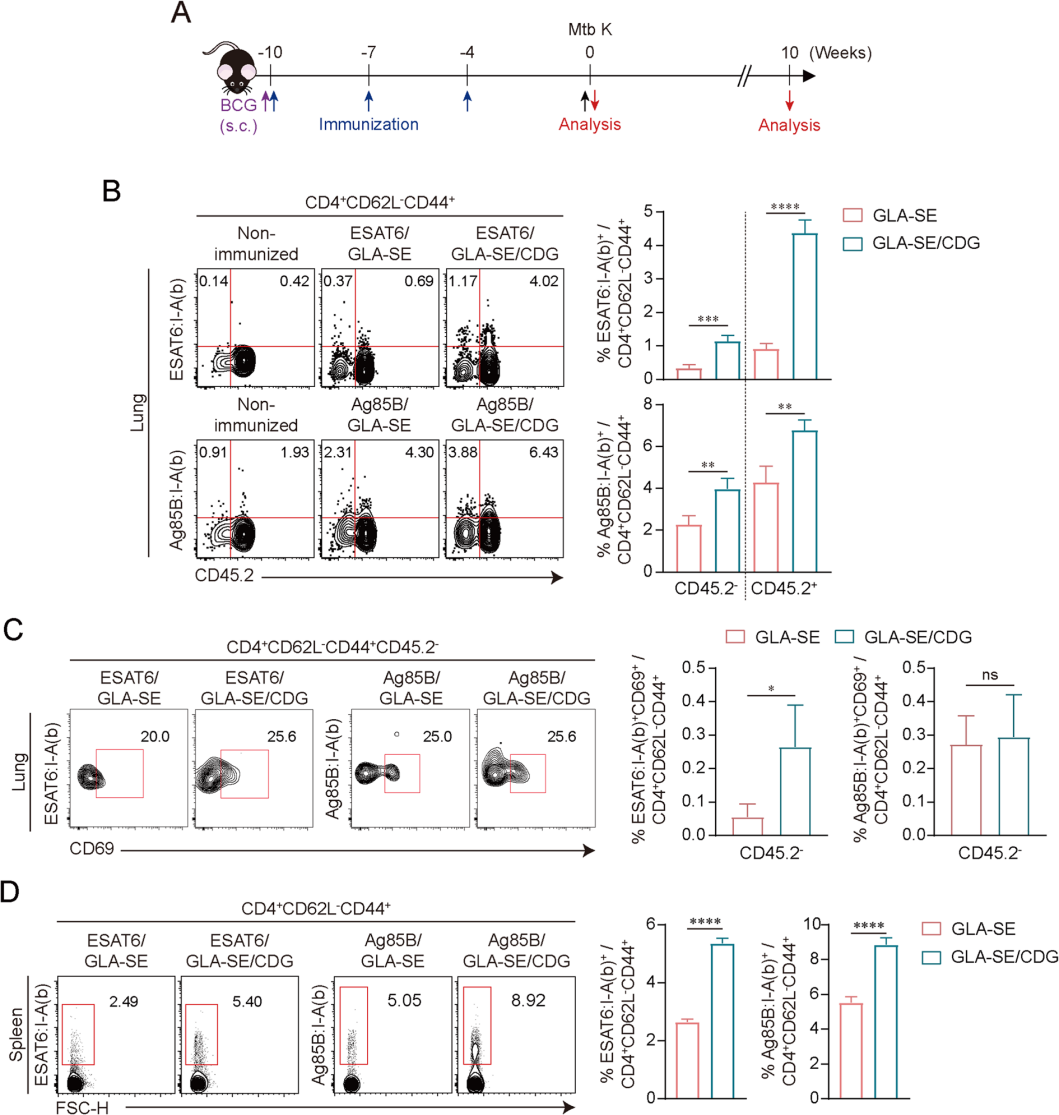
Analysis of antigen (Ag)-specific CD4^+^ T cells induced by either ESAT6 or Ag85B formulated in GLA-SE plus c-di-GMP (CDG) vaccination in the lungs and spleen of immunized mice. **A** Experimental scheme of the in vivo experiment. **B** Representative plots of ESAT6:I-A(b)^+^ or Ag85B:I-A(b)^+^ in CD4^+^ T cells in the lungs from each group (*n* = 4). The numbers in plots (left panel) indicate the frequency of Ag-specific T cells in the lung parenchyma (CD45.2 negative) and intravascular compartments (CD45.2 positive). The frequency of Ag-specific T cells in the lung from each group were summarized in graph (right panel). **C** Representative plots of Ag-specific T cells expressing CD69 in the lung parenchyma (CD45.2 negative) and the frequency of these population was summarized in graph. **D** Representative plots of Ag-specific T cells in spleens from each group. The numbers in plots (left panel) indicate the frequency of Ag-specific T cells in spleen. The frequency of Ag-specific T cells in spleen from each group were summarized in graph (right panel). Graphs show the means ± SD. The data are representative of a single experiment. n.s. not significant, *p < 0.05, **p < 0.01, ***p < 0.001, and ****p < 0.0001 between Ag + GLA-SE- and Ag + GLA-SE/CDG-immunized mice

Next, we employed another TB subunit vaccine component, Ag85B, to determine whether the adjunctive adjuvant effect of CDG is applicable to a different Ag (Fig. 5A). Based on superior pulmonary protection (Fig. 4A and B), we further investigated whether ESAT6 + or Ag85B + GLA-SE/CDG immunization elicited resident memory T cells in the lung, as mucosal localization of CD4^+^ T cells in the lung parenchyma has been associated with protection against Mtb in mice [47, 48]. Using ESAT6_4-17_:I-A(b) or Ag85B_280-294_:I-A(b) tetramer staining together with intravenous labeling with an anti-CD45.2 injection to discriminate cells in the lung vasculature (CD45.2^+^) and lung parenchyma (CD45.2^−^), we observed that immunization including CDG significantly increased ESAT6- or Ag85B-specific CD4^+^ T cells in the lung vasculature and lung parenchyma compared to those from the Ags/GLA-SE-immunized group (Fig. 5B). Specifically, the population of CD45.2^−^ESAT6_4-17_:I-A(b)^+^CD4^+^CD44^+^CD62L^−^CD69^+^ T cells, which were defined as tissue-resident memory (T_rm_)-like cells [49], increased after CDG immunization, whereas the frequency of Ag85B-specific T_rm_-like cells in CDG immunization was comparable to that of the Ag85B + GLA-SE only-immunized group (Fig. 5C). Additionally, in the spleen, both Ags adjuvanted in GLA-SE plus CDG also increased the Ag-specific CD4^+^ T cells prior to infection (Fig. 5D). These results indicate that immunization including CDG commonly increased Ag-specific CD4^+^ T cells against both Ags.

### Superior protection against ultra-low dose Mtb K challenge through the combinatory effect of GLA-SE and CDG

**Fig. 6:**
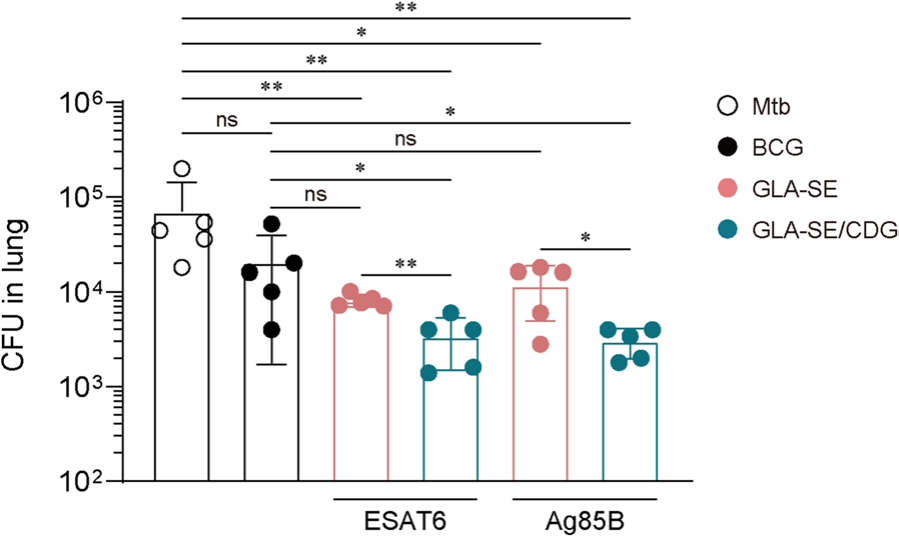
Evaluation of vaccine efficacy in mice immunized with antigen (Ag) + GLA-SE/c-di-GMP (CDG) against ultra-low dose challenge with Mtb strain K. Mtb CFU in both lungs and spleen of each group at 10 weeks post infection were analyzed by enumerating the viable bacteria. Graph shows mean ± SD. The data are representative of a single experiment. Mann–Whitney rank tests were used to compare groups. n.s. not significant, *p < 0.05 and **p < 0.01

Each group of immunized mice was then infected with an ultra-low dose (< 10 CFUs), reflecting physiological inoculum [50]. Thus, Ags/GLA-SE/CDG-immunized mice were further exposed to an ultra-low dose aerosol infection with Mtb K to mimic natural exposure. In terms of pulmonary inflammation, no significant differences were observed among all groups at 10 weeks post Mtb K challenges (Fig. S7). While BCG immunization did not mediate protection, immunizations with Ags/GLA-SE, whether with or without CDG, significantly conferred protection against Mtb K infection compared to Mtb-infected only mice. However, the protective efficacy of Ags/GLA-SE immunized mice was not superior to that of BCG immunization. Improved protections were only mediated by mice immunized with Ags/GLA-SE/CDG. Importantly, immunization of mice with CDG, in combination with either Ag85B/GLA-SE or ESAT6/GLA-SE, further reduced lung bacterial burden compared to Ags/GLA-SE immunized mice (Fig. 6). This suggests that long-term protection was achieved based on the adjunctive adjuvant effect of CDG.

### Subunit vaccine adjuvanted with GLA-SE plus CDG induces Ag-specific CD4^+^ T cells possessing less-differentiated phenotypes

**Fig. 7:**
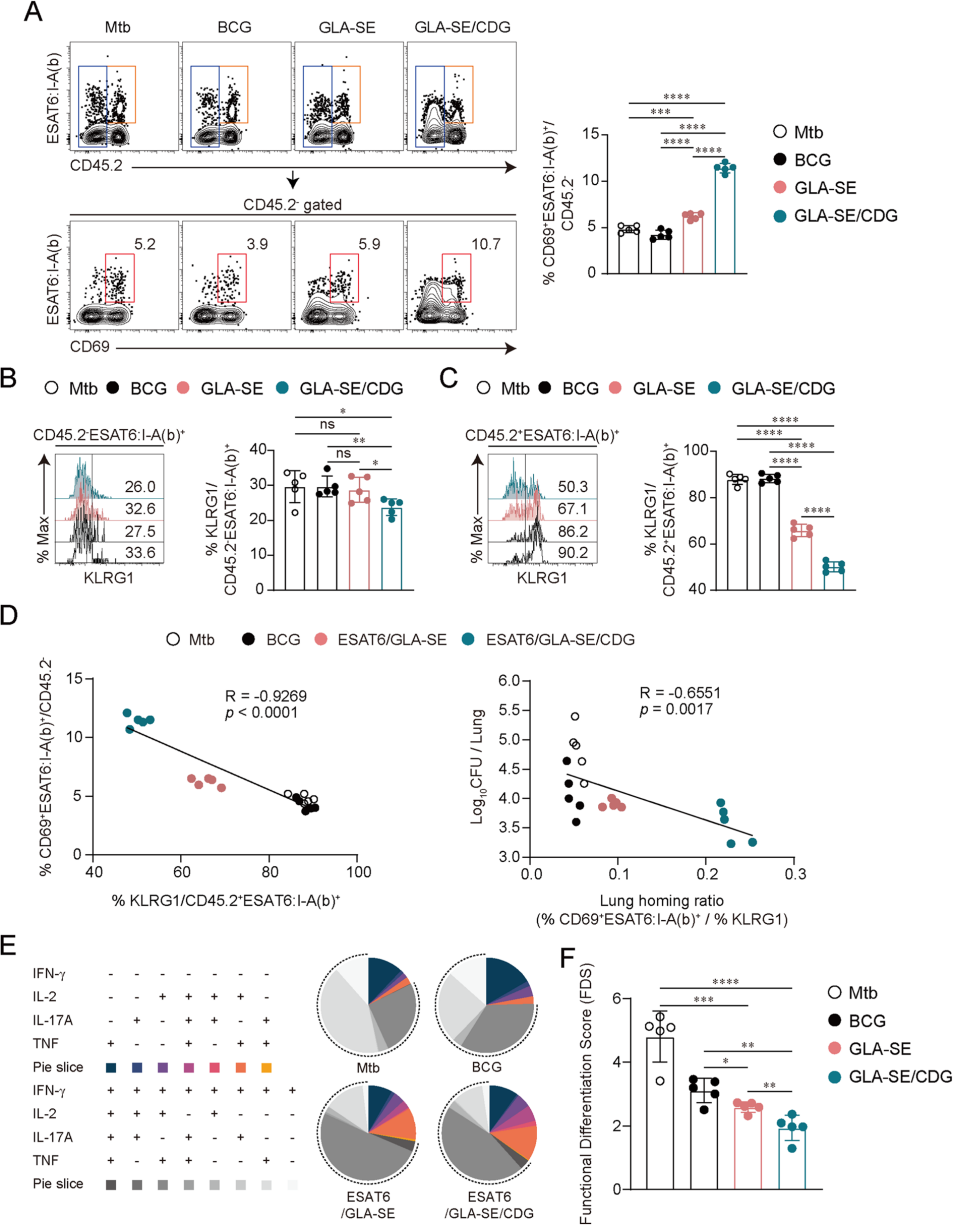
Antigen (Ag)-specific CD4^+^ T cell subsets after ultra-low dose challenge with Mtb strain K. **A** Representative plots of Ag-specific CD4^+^ T cells expressing CD69 in the lung parenchyma (left panel). The frequency of these population was summarized in graph (right panel). **B, C** The frequencies of KLRG1^+^ cells among CD45.2^−^ESAT6:I-A(b)^+^CD4^+^ or CD45.2^−^ESAT6:I-A(b)^+^CD4^+^ T cells were analyzed by flow cytometry and are summarized in the graph (*n* = 4). **D** The correlation between the frequency of CD45.2^+^ESAT6:I-A(b)^+^KLRG1^+^ T cells and CD45.2^−^ESAT6:I-A(b)^+^CD69^+^ T cells in the lung was analyzed (*n* = 5, left panel). The lung homing ratio was calculated as the ratio of the frequency of CD45.2^−^ESAT6:I-A(b)^+^CD69^+^ T cells to the frequency of CD45.2^+^ESAT6:I-A(b)^+^KLRG1^+^ T cells. Then, the correlation between the lung homing ratio and bacterial loads was analyzed (*n* = 5, right panel). **E** Boolean gating analysis of IL-2/IL-17A/TNF/IFN-γ expression of Ag-specific CD4^+^ T cells analyzed 10 weeks post infection. Pies indicate the average proportion of Ag-specific T cells with each combination of cytokine expression. The dotted arches illustrate the fraction of specific CD4.^+^ T cells that produced IFN-γ. **F** The Functional Differentiation Score (FDS) was calculated as the ratio of [IFN-γ producers]: [IFN-γ non-producers] from each subset of mice. Graph shows mean ± SD. The data are representative of a single experiment. The data were analyzed by one-way ANOVA with post hoc Tukey’s test. n.s. not significant, *p < 0.05, **p < 0.01, ***p < 0.001, and ****p < 0.0001

We compared the performance of GLA-SE versus GLA-SE/CDG-including vaccines on CD4^+^ T cell phenotype and functionality after ultra-low dose challenges of Mtb K. Recent studies in mouse models have highlighted that protection is linked to the ability of Mtb-specific CD4^+^ T cells to localize to the lung parenchyma beyond their polyfunctionality [3, 47, 51]. Thus, sustained localization of Mtb-specific T cells within infected lung tissue is likely integral to controlling infection. Notably, killer cell lectin-like receptor subfamily G member 1 (KLRG1) expression has been associated with T cell localization, as terminally differentiated KLRG1^+^CD4^+^ T cells accumulate in the lung vasculature [47, 51]. At 10 weeks post-infection, mice immunized with ESAT6 + GLA-SE/CDG exhibited an increased frequency of Ag-specific CD4^+^ T_rm_-like cells in the lung parenchyma compared to those with ESAT6 + GLA-SE immunization (Fig. 7A). In line with previous reports [47, 51], Ag-specific CD4^+^ T cells in the lung parenchyma displayed low KLRG1 expression across all groups, with its expression further reduced in the ESAT6 + GLA-SE/CDG-immunized group (Fig. 7B). Notably, KLRG1 expression among ESAT6-specific CD4^+^ T cells in the lung vasculature was significantly decreased in ESAT6 + GLA-SE/CDG-immunized mice compared to those with ESAT6 + GLA-SE immunization (Fig. 7C). Furthermore, reduced KLRG1 expression in the lung vasculature was positively correlated with increased Ag-specific T cells in the lung parenchyma. This correlation (the ratio of the frequency of CD45.2^−^ESAT6:I-A(b)^+^CD69^+^ T cells to the frequency of CD45.2^+^ESAT6:I-A(b)^+^KLRG1^+^ T cells) was also associated with improved pulmonary protection against Mtb infection (Fig. 7D). Based on the decreased KLRG1 expression in the ESAT6 + GLA-SE/CDG-immunized group (Fig. 7B and C), we assessed the combinatorial expression of Th1 cytokines in the lung parenchyma, including IFN-γ and TNF co-expression, which indicates terminal differentiation [52]. ESAT6-specific CD4^+^ T cells in the ESAT6 + GLA-SE/CDG-immunized group displayed a less-differentiated phenotype with increased co-expression of TNF and IL-2 without IFN-γ. Simultaneously, ESAT6 + GLA-SE/CDG immunization increased Th17 responses, as the combinatorial expression of IL-17A, a Th17 cytokine, with TNF and/or IL-2 was also increased in the lung parenchyma of ESAT6 + GLA-SE/CDG-immunized mice (Fig. 7E and Fig. S8). Notably, ESAT6-specific CD4^+^ T cells in the ESAT6 + GLA-SE-immunized group had an intermediate level of terminal differentiation between BCG- and ESAT6 + GLA-SE/CDG-immunized groups (Fig. 7E and Fig. S8). We then employed the functional differentiation score (FDS) to quantitatively assess T cell differentiation among groups. Since IFN-γ expression increases as Th1 cells differentiate, a high FDS indicates a response dominated by differentiated T cells, whereas a low score suggests a less-differentiated CD4^+^ T cell population [53–55]. While ESAT6 + GLA-SE immunization significantly reduced the FDS of Ag-specific CD4^+^ T cells in the lung parenchyma compared to Mtb-infected or BCG-immunized groups, Ag-specific T cells in ESAT6 + GLA-SE/CDG-immunized mice had a further reduced FDS score, indicating that the inclusion of CDG induced less differentiation of Ag-specific T cells than those induced by GLA-SE-only immunization (Fig. 7F). Unlike ESAT6, the phenotypes of Ag85B-specific CD4^+^ T cells were not further elucidated, as these T cells were scarcely detected in the lung across all groups (Fig. S9). Nevertheless, we observed a common increase in IL-17A production in the lungs of Ag85B or ESAT6 + GLA-SE/CDG-immunized mice upon ex vivo stimulation with Ag85B or ESAT6, respectively, compared to the Ag85B or ESAT6 + GLA-SE immunized group (Fig. S10). Additionally, this increased production was not observed in the lungs of Ag85B or ESAT6 + GLA-SE/CDG-immunized mice prior to Mtb infection (Fig. S11), suggesting that infection might trigger Th17 responses in CDG-immunized mice. Overall, these data demonstrate that the co-administration of CDG induces accumulation of Ag-specific T cells in the lung vasculature with reduced KLRG1 expression, less-differentiated CD4^+^ Th1 cells, and increased Th17 responses following pulmonary Mtb infection.

## Discussion

The rational design of improved and advanced vaccines requires a deep understanding of the innate immune system and how it effectively mediates the induction of protective Ag-specific adaptive immune responses. Including an adjuvant as an immunity-enhancing component in a vaccine formulation can improve the response to the combined antigen. Understanding its mechanism will aid in developing an agent capable of inducing an appropriate adaptive immune response against pathogens. In the current study, we employed CDG as a potent vaccine adjuvant against Mtb Beijing clinical isolates, formulated with model Ags, ESAT-6 or Ag85B. We found that CDG displayed adjuvanticity on its own, ultimately creating enhanced protective immunity when combined with well-established TLR4 adjuvants. This collaborative effect was achieved through the induction of less-differentiated Th1 cells in lung compartments and increased Th17 responses during chronic infections with Mtb. Given that CDG-adjuvanted Ags have been reported to mediate protection against Mtb infection [41, 42, 56, 57], in this study, we further explored the potential use of CDG in combination with well-established adjuvants.

The combined activation of multiple PRRs can elicit synergistic effects that induce robust adaptive immune responses [58–60]. Phase 2 clinical studies have described that a vaccine based on the tumor antigen MAGE-3, when combined with MPL, QS-21, CpG, and liposomes, displayed better protective efficacy compared to the same antigen combined with MPL, QS-21, and oil-in-water emulsions [61]. These results further support the rationale that cooperation from heterogeneous activation can be used to improve vaccine efficacy. Based on this concept, we decided to use adjuvants associated with both TLR-independent and TLR-dependent pathways through the combined use of MPL or GLA and CDG. Cyclic dinucleotides formulated in a protein subunit vaccine demonstrated the induction of long-lasting protective immunity, which correlated with Ag-specific Th1 and Th17 immune responses to Mtb in a mouse model [41, 42]. Additionally, Ning et al. recently showed that a subunit ESAT6-based vaccine formulated with c-di-AMP, a STING activator, resulted in a significant reduction of Mtb burdens in the lungs and spleens when delivered via the intranasal route [57]. Although the route of immunization differed from previous studies, the current study also confirmed the adjuvant potential of CDG, providing long-term protection at levels comparable to MPL against Mtb Beijing clinical isolate infection when delivered intramuscularly. Moreover, CDG in combination with TLR4 adjuvants (MPL or GLA) mediated durable protection against infections by two Mtb Beijing clinical isolates. This was evidenced by bacterial reductions and amelioration of pulmonary inflammation, contributing to additional protective benefits. It is widely acknowledged that a primary goal of TB vaccines, as supported by clinical studies to date, is to elicit polyfunctional Ag-specific Th1 responses for protection against Mtb infection [62, 63]. In our data, Ag-specific CD4^+^ T cells with polyfunctional properties co-producing IFN-γ, TNF, and/or IL-2 were significantly increased in both the lungs and spleens of mice immunized with either ESAT6 + MPL/CDG/DDA or ESAT6 + GLA-SE compared to those immunized without CDG, suggesting that the adjunctive adjuvant CDG enhanced polyfunctional Th1 responses.

Recently, beyond the multifunctionality of vaccine-specific CD4^+^ T cells, T_rms_ have demonstrated an ability to enhance the adaptive immune response to various pathogens and improve infection control [64, 65]. Thus, we next evaluated the mucosal localization of Ag-specific T cells after immunization with the well-characterized TB subunit antigens Ag85B and ESAT6 using in vivo labeling with intravenously injected fluorescent antibodies. The inclusion of CDG resulted in an increased induction of Ag85B- or ESAT6-specific CD4^+^ T cells in the lung parenchyma, vasculature, and spleen. Notably, ESAT6-specific T_rm_ expressing CD69 in the lung parenchyma was more pronounced with the addition of CDG compared to Ag85B. Although CD69 or CD103 have been reported to be important for tissue retention, other markers such as CD49a could also play a role in T cell positioning in the lung parenchyma [66, 67]. Given that Ag85B-specific T cells also increased with CDG addition in the lung parenchyma post-immunization, it would be worthwhile to further analyze other tissue retention markers in future work. Additionally, the extent of T_rm_ induction elicited by CDG appears somewhat marginal compared to previous reports [41, 42], suggesting that these observations could be due to the route of immunization. To induce lung-localized Ag-specific T cells, vaccines are often delivered via mucosal routes such as intranasal or airway vaccinations [68, 69]. However, intramuscular immunization has also been reported to elicit mucosal immune responses in the intestines or lungs [44]. Nonetheless, the inclusion of CDG with both antigens mediated significant long-term pulmonary protection upon an ultra-low dose challenge with the Mtb Beijing clinical isolate K.

Based on the improved protection achieved with the inclusion of CDG, we further explored the role of T cells following Mtb infection. T cells with low to intermediate differentiation along the Th1 axis are known to offer significant protection against Mtb infection in mouse models due to their superior ability to migrate to the lung parenchyma [47, 70]. Consistent with pre-infection observations, sustained and expanded ESAT6:I-A(b)^+^CD69^+^ T cells in the lung parenchyma (CD45.2^−^) were confirmed upon CDG addition at 10 weeks post-Mtb K infection. Importantly, in the lung vasculature (CD45.2^+^) of ESAT6 + GLA-SE/CDG-immunized mice, ESAT6:I-A(b)^+^ T cells displayed the lowest level of KLRG1 expression among the groups. KLRG1 is associated with terminal differentiation of T cells, and its expression on CD4^+^ T cells is linked to poor migration into the lung, leading to accumulation in the vasculature and ultimately failing to inhibit Mtb growth [47, 48, 52, 70–72]. Therefore, effective control of Mtb is facilitated by Th1 cells that are not terminally differentiated, allowing them to migrate into the parenchyma of the Mtb-infected lung [48]. Moreover, it has been reported that ESAT6 was only Ag among screened immunodominant Ags which is crucial for long-term protection especially when rescued from terminal differentiation of ESAT6-specific T cells targeted by vaccination [53]. Our data also showed that decreased KLRG1 expression on ESAT6-specific CD4^+^ T cells in the lung vasculature was positively correlated with increased ESAT6-specific CD4^+^ T cells expressing CD69 in the lung parenchyma. This correlation was also associated with improved bacterial control, although this is not direct evidence. In line with reduced KLRG1 expression, notably, ESAT6-specific CD4^+^ T cells in the lung parenchyma from ESAT6 + GLA-SE/CDG-immunized mice exhibited less-differentiated Th1 phenotypes with a low FDS score and increased Th17 responses after Mtb infection. Th17 responses have been reported to enhance control of Mtb with cooperation of Th1 responses [48, 73]. Since increased Th17 responses by CDG inclusion were not observed post-immunization, this may be partially due to intraclonal competition for Ags, which occurs when Mtb-specific T cells switch from a Th1 to a Th17 response by limiting terminal differentiation in the presence of an abnormally high number of T cells with identical TCRs [71]. It might be conceivable that the accelerated and robust increase of Ag-specific Th1 cells in both the lung and spleen, due to CDG addition to immunization, could skew towards Th17 cells following pulmonary Mtb infection. Collectively, CDG-derived less-differentiated CD4^+^ T cells might be a crucial component of long-term vaccine-induced immunity against Mtb infection.

Our current study has certain limitations that warrant further consideration. Firstly, although it was observed that increased protection was mediated by CDG inclusion during immunization, our vaccine experiment which performed after confirming CDG’s independent adjuvanticity lacked a group of CDG-only immunized mice. To verify that observed effects are not solely due to CDG, including this group in future studies would be required for assessing CDG as an optimal adjunctive adjuvant with other adjuvants. For example, to evaluate this, the Loewe definition of additivity, which classifies the interaction as synergistic (D < 1), additive (D = 1), or antagonistic (D > 1) could be potentially employed [74, 75]. Based on concentration–response curves generated from vaccine formulations with different concentration combinations of each agonist, we might provide more compelling evidence to clarify whether the enhancement observed from combining agonists equaled the cumulative effects of each agonist individually (additive), or if the TLR4 agonist and CDG worked together to synergistically mediate improved protection in future investigations. Secondly, Ag85B-specific T cells were scarcely detected after Mtb infection, in contrast to ESAT6-specific T cells, although both types of T cells were observed prior to Mtb infection. This suggests that differential antigenic effects of ESAT6 and Ag85B might be elicited during infection. The priming of T cells is influenced by the affinity, quantity, and duration of antigen exposure, as these factors can affect the differentiation of subsequent memory responses [76, 77]. In addition, CD4^+^ T cells’ capacity to mediate protection is likely determined by the specific Mtb Ags they recognize, with each Ag exhibiting distinct expression patterns across various stages of infection [54]. Initially increased CD4^+^ T cell responsiveness to Ag85B, which is involved in the synthesis of mycobacterial cell wall [78], decreases due to reduced expression of this Ag during chronic phase of infection [54, 79, 80], thereby restricting their capacity to control Mtb infections. On the other hand, ESAT6, which is actively secreted by Mtb [81], was recognized by T cells throughout Mtb infection. This results in ESAT6-specific T cells becoming terminally differentiated and functionally exhausted due to chronic Ag stimulation [54]. These findings may explain our observation of the scarce detection of Ag85B-specific T cells, as T cells were analyzed during the chronic phase of infection. Given that enhanced protection was achieved through Ag85B + GLA-SE/CDG immunization, future studies need to analyze Ag85B-specific T cells during the acute phase of infection to clarify the effect of CDG on these T cells and their impact on the early control of Mtb. Nevertheless, CDG inclusion might display potential as an adjunctive adjuvant for TB vaccines, as ESAT6 + GLA-SE/CDG immunization rescued ESAT6-specific T cells from terminal differentiation during chronic infection, accompanied by improved protection. Regardless, these findings, including our observations, have important implications: selecting a single Mtb Ag for developing an improved TB vaccine might not be optimal, and a deep understanding of differential Mtb Ag availability according to the infection stage is required in the rational design of TB vaccines. Based on this, for broad application of the effectiveness of CDG, experimental approaches using additional Ags need to be addressed in our future studies. Furthermore, we observed that the production of IL-17A was commonly increased in the lungs of the ESAT6- or Ag85B-GLA-SE/CDG-immunized groups upon stimulation with ESAT6 or Ag85B, respectively. Therefore, analyzing the acute phase and challenges with a conventional dose of Mtb should be considered in future studies to investigate Th1/Th17 skewing and the acquisition of less-differentiated phenotypes in Ag85B-specific T cells as a common protective signature mediated by CDG. Thirdly, an adoptive transfer experiment would be valuable to support the hypothesis that CDG-derived ESAT6^+^CD4^+^KLRG1^lo^ T cells in the lung vasculature (CD45.2^+^) retain enhanced homing capacity to the lung parenchyma. Nonetheless, in line with previous reports [47, 71], we consistently observed that CD4^+^ T cells with increased expression of KLRG1 in Mtb-infected only mice in the lung vasculature inversely correlated with T cells in the lung parenchyma. In line with this, further study using FTY720, which inhibits cell trafficking from secondary lymphoid organs to inflamed sites, should be helpful in understanding whether CDG-derived T_rm_, less-differentiated T cells retaining the capacity to traffic into the parenchyma, or orchestration could be determining factors for their pulmonary protective capacity. Fourthly, in the current study, we observed the improved efficacy conferred by the inclusion of CDG based on experiments conducted once. This limitation affects the potential of CDG as an adjunctive adjuvant for TB vaccines. Although enhanced protection mediated by the inclusion of CDG in two different TLR4 adjuvants with two individual subunit Ags was observed against Mtb clinical isolates, repeated experimental approaches should be conducted to warrant its practical use. Lastly, given that the rising incidence of TB among adults who have received the BCG might be linked to the diminishing effectiveness of the vaccine over time [82], enhancing BCG immunity in this age group represents a significant conceptual breakthrough in the development of TB vaccines. However, latent TB in TB endemic region could act as a considerable source of mycobacterial sensitization, affecting the efficacy of BCG [83]. Additionally, selecting persistently expressed TB vaccine Ags and using the same Ags as a booster approach in these regions might induce terminally differentiated T cells with dysfunction [54]. Although Ags + GLA-SE/CDG immunization conferred superior protection compared to BCG against primary infection, this improved efficacy might be because it was tested in mice that are naïve to mycobacteria. Thus, testing the efficacy of our vaccine in an Mtb post-exposure model, with careful consideration of optimizing CDG-containing subunit vaccine formulations, should be required for use in the next generation of TB vaccines.

## Conclusions

**Fig. 8:**
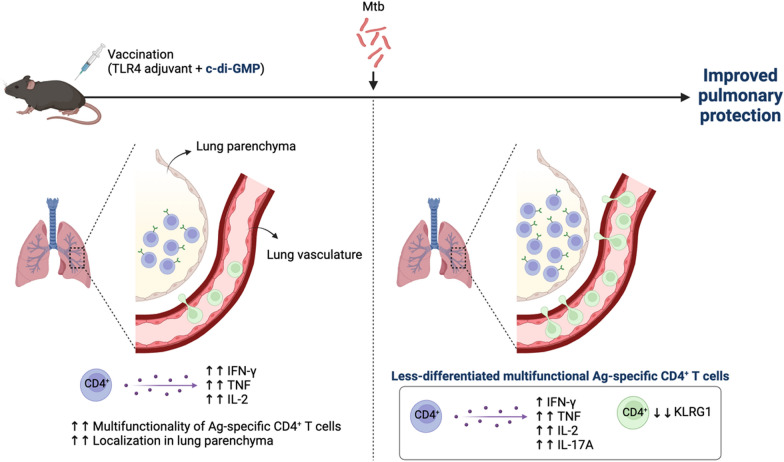
Graphical abstract of our experiments. The integration of Ag in TLR4 and CDG adjuvant formulations boosted Th1-biased, multifunctional Ag-specific CD4^+^ T cells in the lungs and spleen. The inclusion of CDG increased the localization of these T cells to the lung mucosa, and the presence of Ag-specific CD4^+^ T cells in the lung blood vessels. Following Mtb infection, the addition of CDG reduced the expression of KLRG1 in Ag-specific CD4.^+^ T cells, resulting in less-differentiated phenotypes and significantly lower bacterial loads compared to groups that were not immunized, received BCG, or were immunized with Ag/TLR4 without CDG. (Created in https://BioRender.com)

Overall, our study demonstrated that widely used TB subunit vaccine Ags adjuvanted with TLR4 (MPL and GLA) and STING (CDG) agonists cooperatively and effectively elicited robust cellular immune responses, ultimately providing better long-term protective efficacy compared to using each adjuvant individually (Fig. 8). The rational design of this adjuvant combination holds significant potential, especially for TB vaccines incorporating immunodominant Ags, and will facilitate the development of effective future vaccines.

## Supplementary Information

Additional file 1.

## Data Availability

All other data are available in the article and its Supplemental files or from the corresponding author upon reasonable request.

## Abbreviations

Ags: Antigens
BCG: Bacillus Calmette–Guérin
CDG: C-di-GMP
CFU: Colony forming unit
cGAMP: Cyclic-di-GMP-AMP
cGAS: Cyclic-di-GMP-AMP synthase
DDA: Dimethyldioctadecylammonium
ELISA: Enzyme-linked immunosorbent assay
FDS: Functional differentiation score
GLA-SE: Glucopyranosyl lipid adjuvant-stable emulsion
H&E: Hematoxylin and eosin
IFN: Interferon
IL: Interleukin
IRF3: Interferon regulatory factor 3
KLRG1: Killer cell lectin-like receptor subfamily G member 1
MPL: Monophosphoryl lipid A
Mtb: *Mycobacterium tuberculosis*
NLRs: Nucleotide-binding oligomerization domain-like receptors
NF-B: Nuclear factor kappa B
PAMPs: Pathogen-associated molecular patterns
PRR: Pattern recognition receptors
SPF: Specific pathogen-free
STING: Stimulator of interferon genes
TB: Tuberculosis
TBK1: Tank-binding kinase-1
TLR: Toll-like receptors
TNF: Tumor necrosis factor
T_rm_: Tissue-resident memory T cell
