# Supplementary material for: Adjunctive beneficial effect of c-di-GMP, a STING agonist, in enhancing protective efficacy of TLR4-adjuvanted tuberculosis subunit vaccine formulations
Source: J Biomed Sci. 2025 May 26;32:52. doi: 10.1186/s12929-025-01144-8 (PMC12105139; doi:10.1186/s12929-025-01144-8)
Supplement: Supplementary file 1 — Additional file 1. [file 12929_2025_1144_MOESM1_ESM.docx]

**Adjunctive beneficial effect of c-di-GMP, a STING agonist, in enhancing protective efficacy of TLR4-adjuvanted tuberculosis subunit vaccine formulations**

Kee Woong Kwon^1,2^, Eunsol Choi^2^, Hagyu Kim^2^, Hyeong Woo Kim^1^, Sangwon Choi^2^, Seunghyun Lee^2^, Sang-Jun Ha^3,4^, and Sung Jae Shin^2,5*^

^1^Department of Microbiology and Convergence of Medical Science, College of Medicine, Gyeongsang National University, Jinju, 52727, Republic of Korea, ^2^Department of Microbiology, Graduate School of Medical Science, Brain Korea 21 Project, Yonsei University College of Medicine, Seoul 03722, South Korea, ^3^Department of Biochemistry, College of Life Science & Biotechnology, Yonsei University, Seoul 03722, South Korea, ^4^Brain Korea 21 (BK21) FOUR Program, Yonsei Education & Research Center for Biosystems, Yonsei University, Seoul, 03722, Republic of Korea, ^5^Institute for Immunology and Immunological Disease, Yonsei University College of Medicine, Seoul, 03722, South Korea

* Correspondence:

Sung Jae Shin, Department of Microbiology, Graduate School of Medical Science, Brain Korea 21 Project, Yonsei University College of Medicine, Seoul 03722, South Korea., Tel.: (82) 2-2228-1813, Fax: (82) 2-2-392-9310; E-mail: [sjshin@yuhs.ac](mailto:sjshin@yuhs.ac)**Supplementary Figures**

**
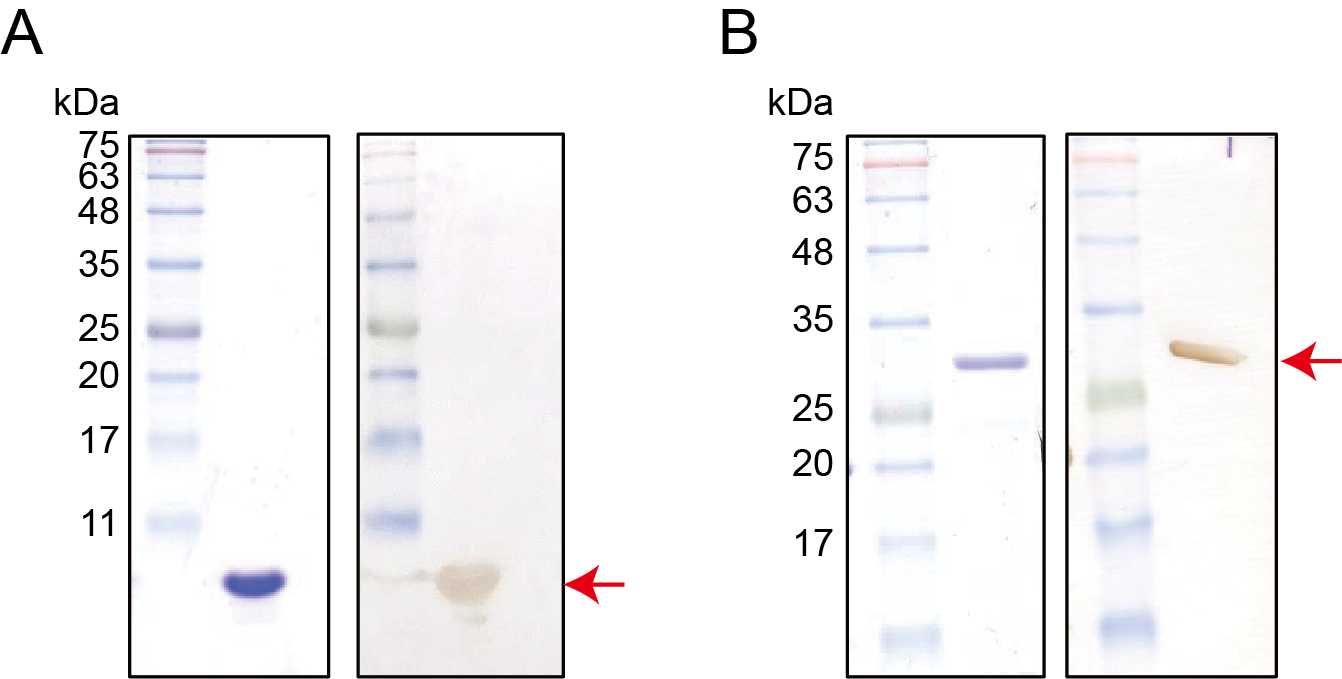
**

**Supplementary Figure 1. Purification of recombinant proteins. A** and **B** Purified recombinant ESAT6 (A; 9.9 kDa) and Ag85B (B; 32.0 kDa) proteins were analyzed by SDS-PAGE with Coomassie blue staining (left) and immunoblot analysis using anti-histidine antibodies (right).


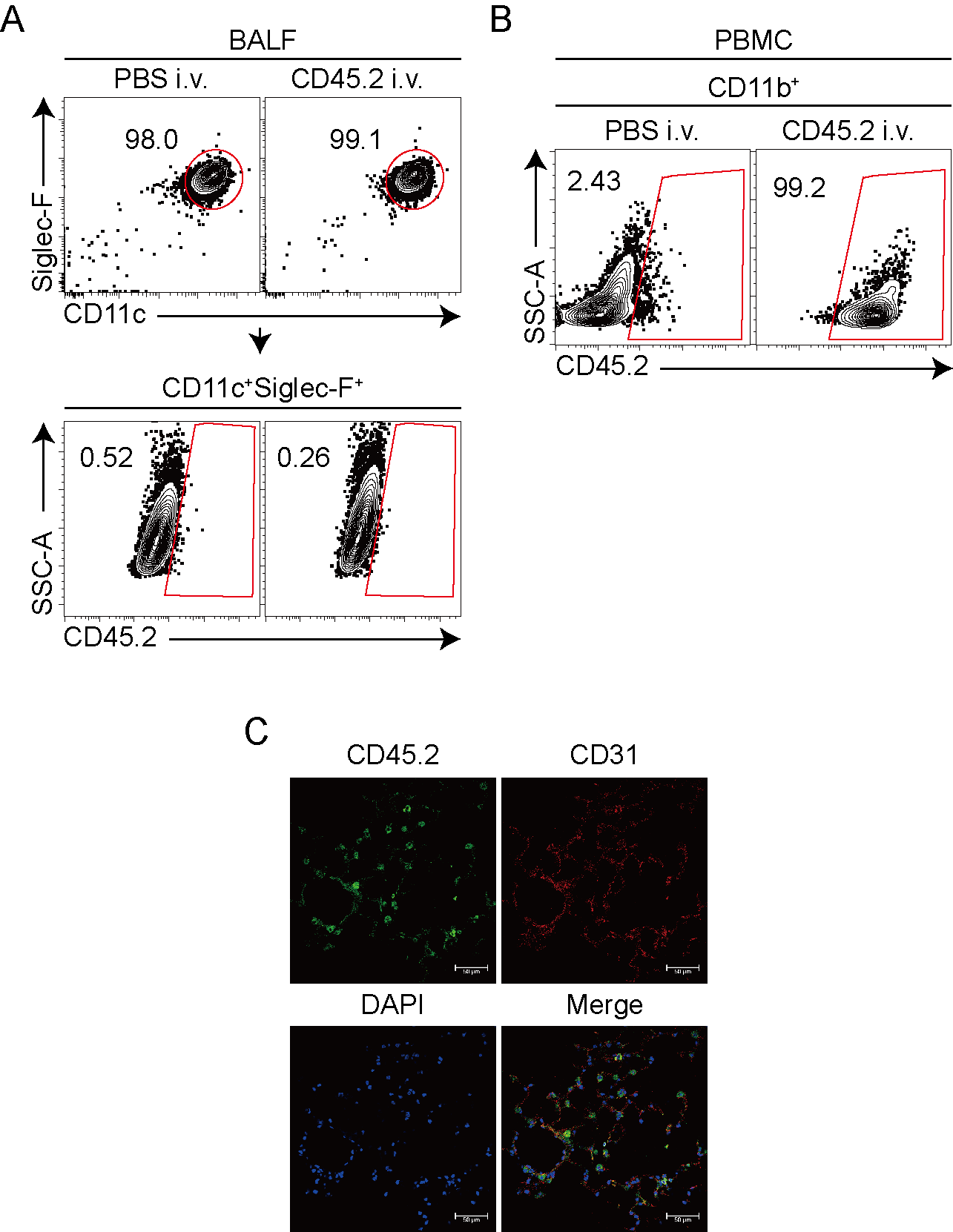


**Supplementary Figure 2. *In vivo* intravascular labeling of the lung. A** and **B** After intravenous injection of Alexa594-conjugated anti-CD45.2, BALF (A) and PBMCs (B) were analyzed as negative and positive controls, respectively. **C** Immunofluorescence staining of the lung coupled with DAPI (blue) after co-injection of anti-CD45.2 (green) and anti-CD31 (red) (20X: scale bar = 50 μm).

**
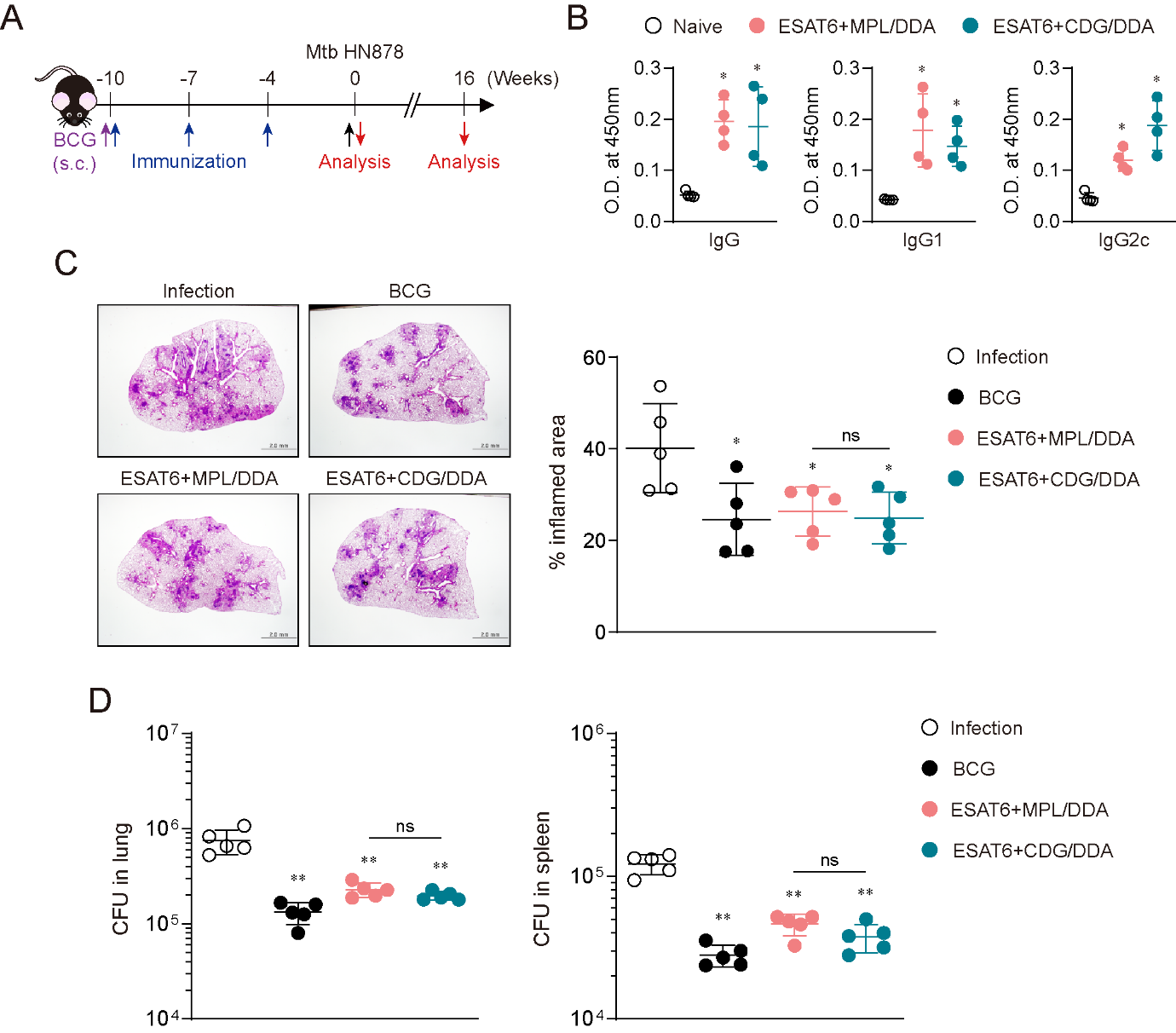
**

**Supplementary Figure 3. Evaluating adjuvant potential of c-di-GMP (CDG) formulated together with ESAT6. A** Scheme of the experimental design for evaluating the adjuvant potential of CDG by using an ESAT6 model antigen (Ag). **B** Individual mouse sera (n = 4) were isolated at 4 weeks after the final immunization, and ESAT6-specific IgG, IgG1, and IgG2c levels were measured with an ELISA. **C** H&E staining of lungs of each group (10X: scale bar = 2.0 mm). The experimental results indicated the percentages of inflamed area and are described by dot plots. **D** Mtb CFU in both lungs and spleen of each group (n = 5) at 16 weeks post infection were analyzed by enumerating the viable bacteria. Graph shows mean ± SD. The data are representative of a single experiment. Mann–Whitney rank tests were used to compare groups. n.s. not significant, *p < 0.05 and **p < 0.01.


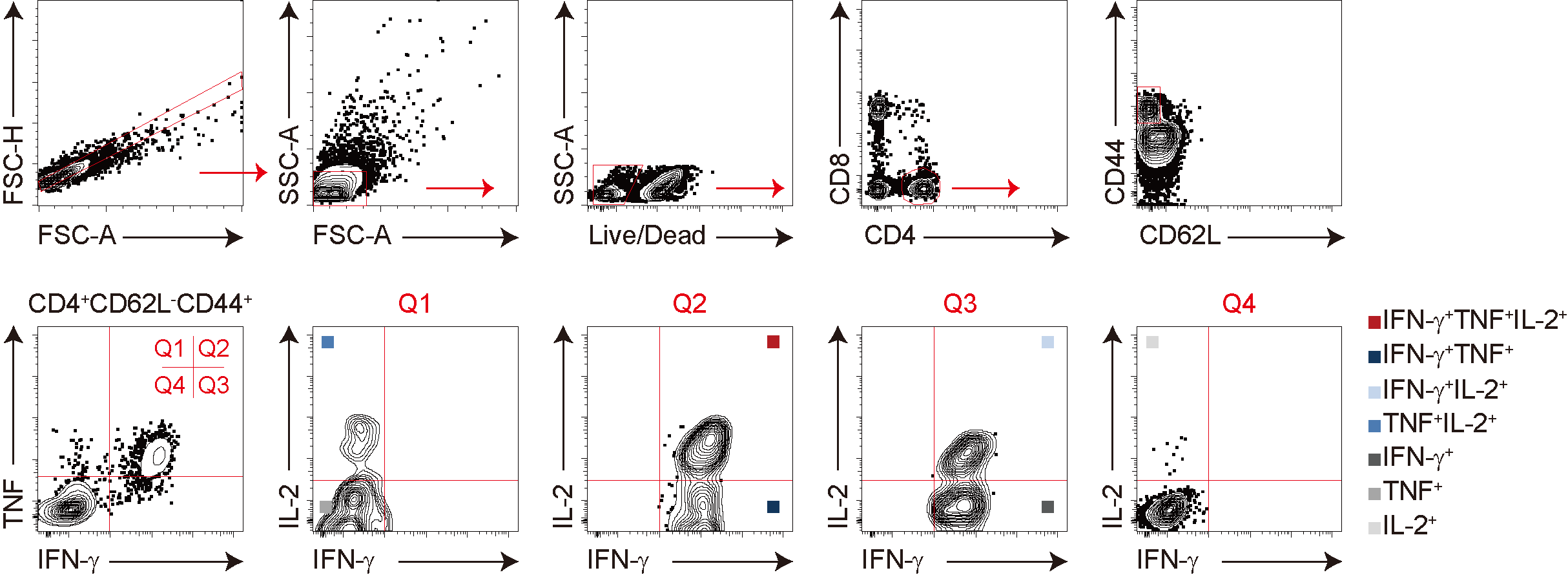


**Supplementary Figure 4. Gating strategy for dissecting multifunctional T cell population.** All samples stained for surface and intracellular cytokines were gated based on forward scatter (FSC) and side scatter (SSC). T cells were gated based on CD4 expression. Specific staining for intracellular cytokines is displayed using CD4^+^CD44^+^CD62L^-^ T cell gating.


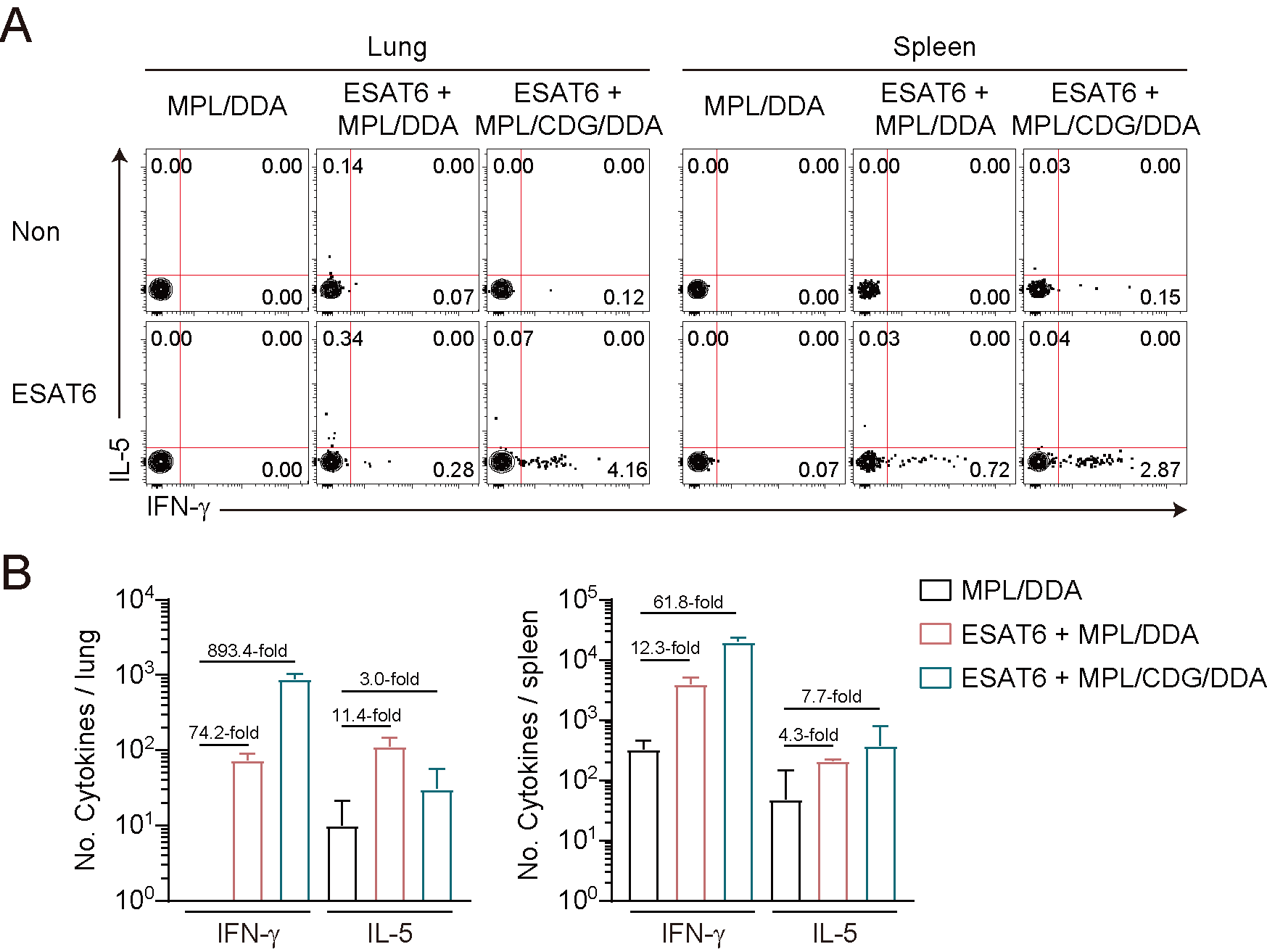


**Supplementary Figure 5. Analysis of CD4^+^ Th2 responses in the lungs and spleen of ESAT6+MPL/CDG/DDA-immunized mice. A** Representative plots of IL-5^+^ and IFN-γ^+^ in CD4^+^ T cells. The numbers in plots indicate the frequency of IL-5^+^IFN-γ^+^, IL-5^+^IFN-γ^−^, and IL-5^−^IFN-γ^+^. **B** The number of IL-5^+^ and IFN-γ^+^ CD4^+^ T cells in lungs were summarized in graphs with fold-change. The data are representative of a single experiment.


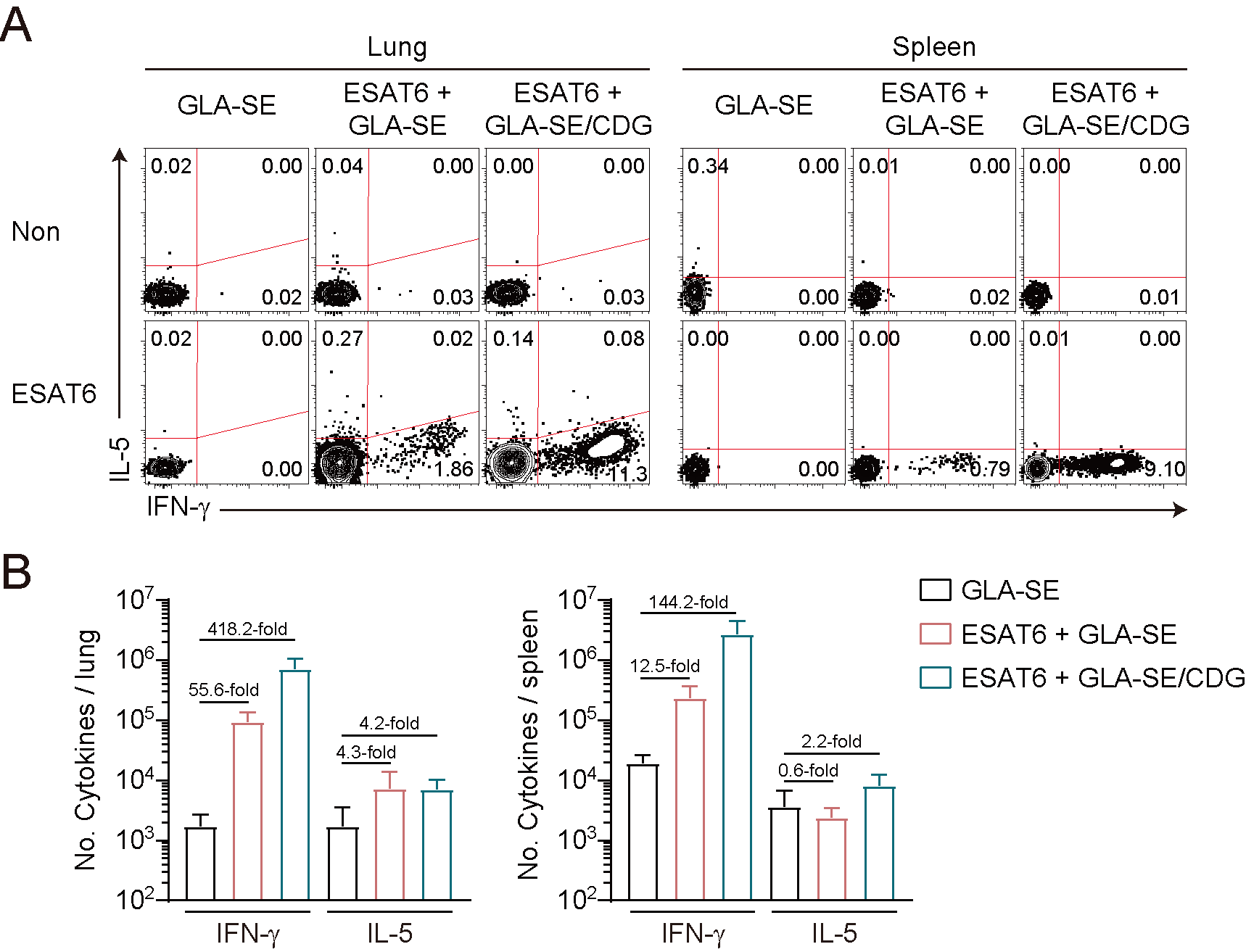


**Supplementary Figure 6. Analysis of CD4^+^ Th2 responses in the lungs and spleen of ESAT6+GLA-SE/CDG-immunized mice. A** Representative plots of IL-5^+^ and IFN-γ^+^ in CD4^+^ T cells. The numbers in plots indicate the frequency of IL-5^+^IFN-γ^+^, IL-5^+^IFN-γ^−^, and IL-5^−^IFN-γ^+^. **B** The number of IL-5^+^ and IFN-γ^+^ CD4^+^ T cells in lungs were summarized in graphs with fold-change. The data are representative of a single experiment.


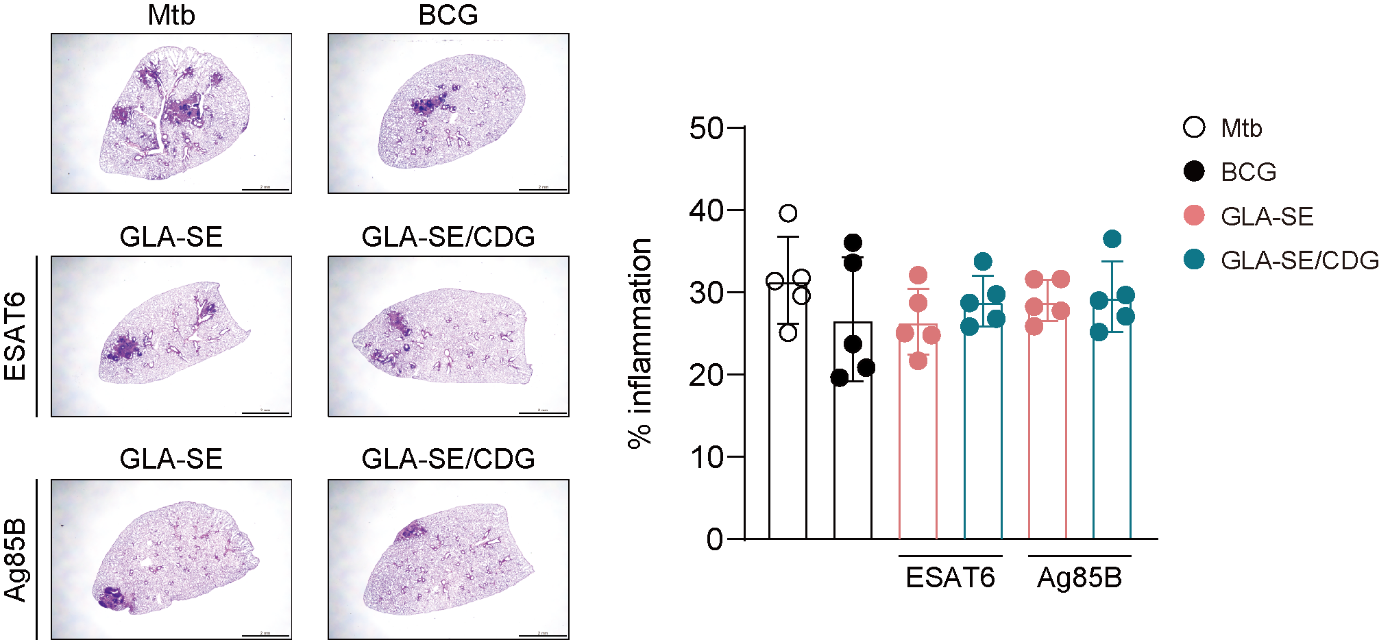


**Supplementary Figure 7. Pulmonary inflammation in mice 10 weeks after challenge with Mtb K.** H&E staining of lungs of each group (*n* = 5; 10X: scale bar = 2.0 mm) at 10 weeks post Mtb infection. The experimental results indicated the percentages of inflamed area and are described by dot plots. Graph shows mean ± SD. The data are representative of a single experiment. Mann–Whitney rank tests were used to compare groups. No significant differences between groups were found.


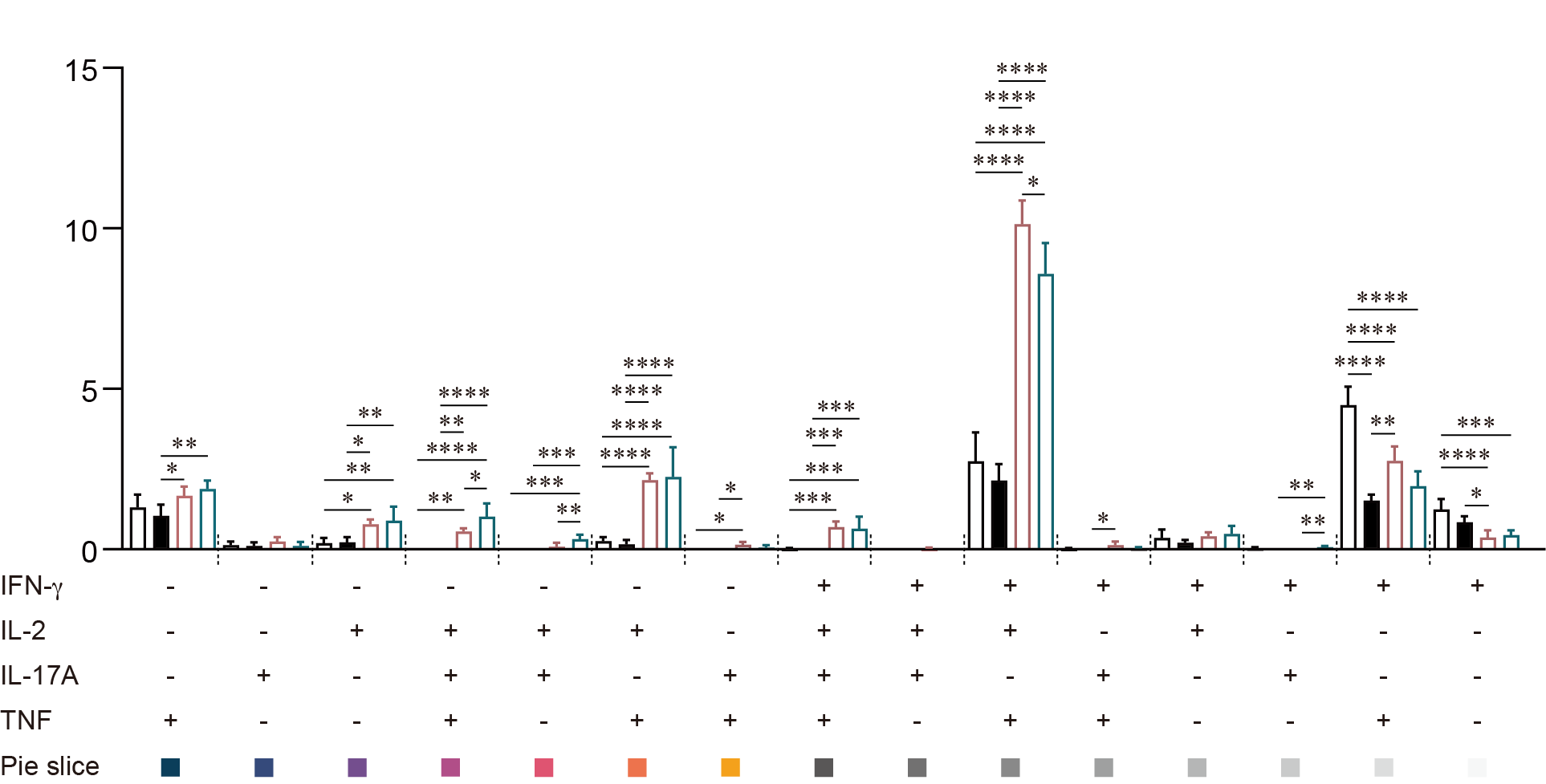


**Supplementary Figure 8.** **Analysis of CD4^+^ T cell responses induced by ESAT6+GLA-SE in the presence or absence of c-di-GMP (CDG) immunization in the lungs after ultra-low dose challenge with Mtb strain K.** ESAT6-stimulated lung cells from each subset of mice (*n* = 5) were assessed based on the frequency of CD4^+^CD44^+^ T cells with different patterns of cytokine production and presented as bar graph. Graph shows mean ± SD. The data are representative of a single experiment. The data were analyzed by one-way ANOVA with *post hoc* Tukey’s test. n.s. not significant, *p < 0.05, **p < 0.01, ***p < 0.001, and ****p < 0.0001.

**
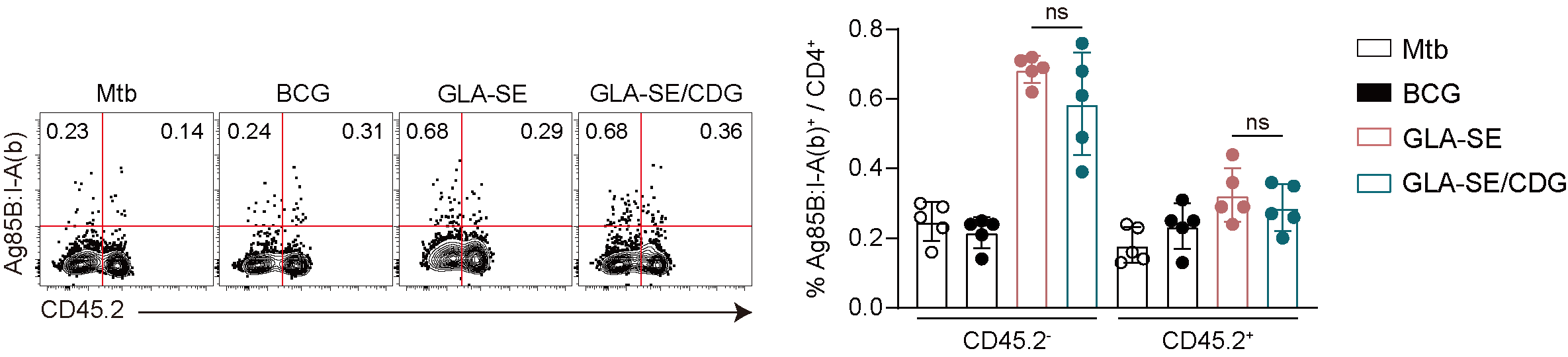
**

**Supplementary Figure 9. Analysis of Ag-specific CD4^+^ T cells in the lungs of Ag85B+GLA-SE/CDG-immunized mice following the Mtb K infection. a** Ten weeks post Mtb infection, with the use of Ag85B-tetramer, Ag-specific CD4^+^ T cells were evaluated in the lung parenchyma (CD45.2 negative) and intravascular compartments (CD45.2 positive). The graphs show the frequency of CD4^+^CD62L^-^CD44^+^Ag85B:I-A(b)^+^ T cells in the parenchyma and intravascular compartments. All bar graphs show the means ± SD of 5 samples. The data are representative of a single experiment. Statistically significant differences between the groups were determined using an unpaired Student’s *t* test. **p < 0.01.

**
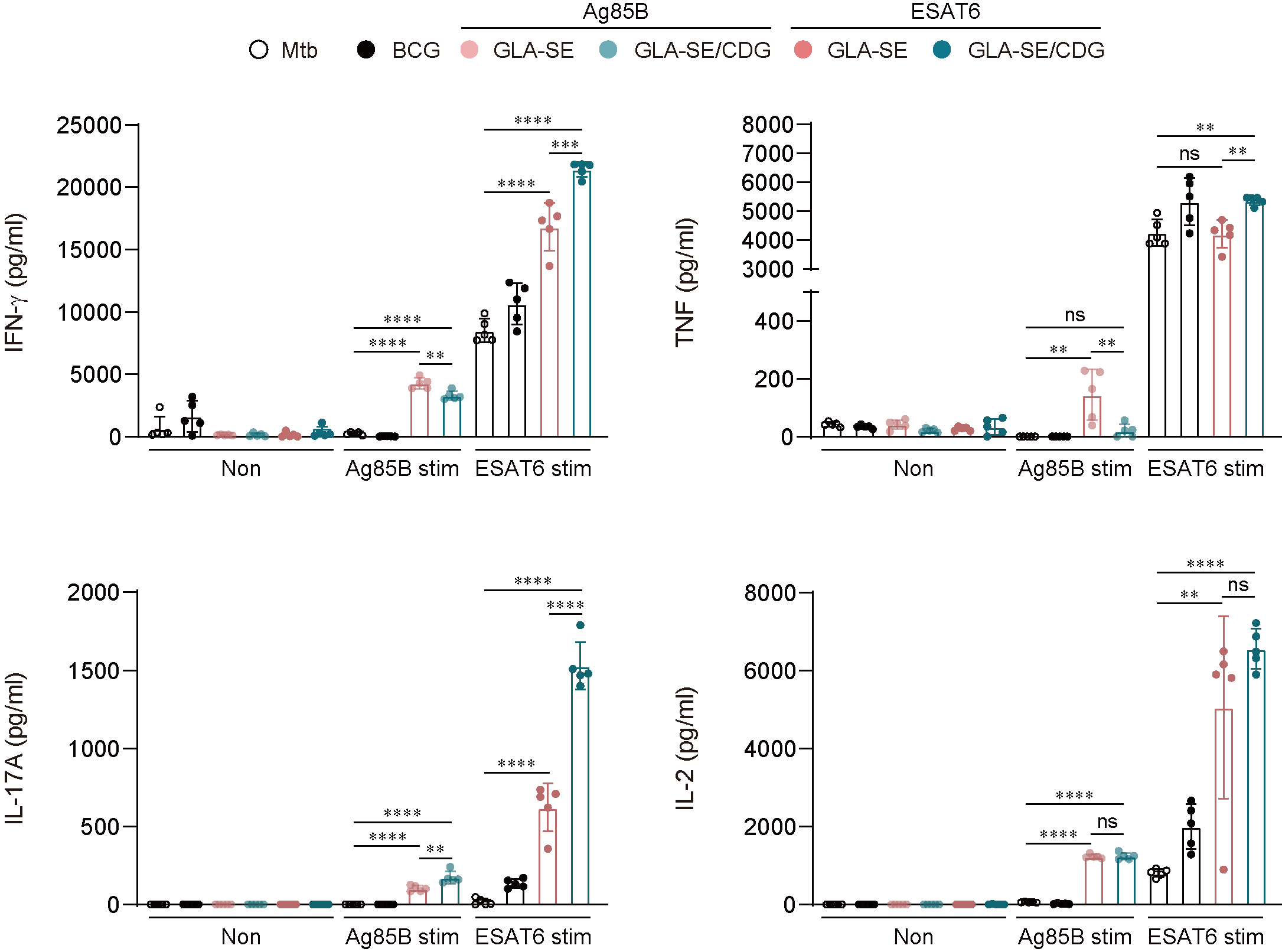
**

**Supplementary Figure 10. Comparative cytokine production profiles in Ag+GLA-SE/CDG-immunized mice upon Ag stimulation at 10 weeks post Mtb K infection.** Levels of IFN-γ, TNF, IL-2, and IL-17A secreted by lung cells in all treatment groups in response to ESAT6 (1 μg/ml) or Ag85B (1 μg/ml) stimulation as detected by ELISA. All bar graphs show the means ± SD of 5 samples. The data are representative of a single experiment. n.s. not significant, **p < 0.01, and ****p < 0.0001 compared to Mtb-infected only mice. n.s. not significant, **p < 0.01, ***p < 0.001, and ****p < 0.0001 between Ag+GLA-SE- and Ag+GLA-SE/CDG-immunized mice.


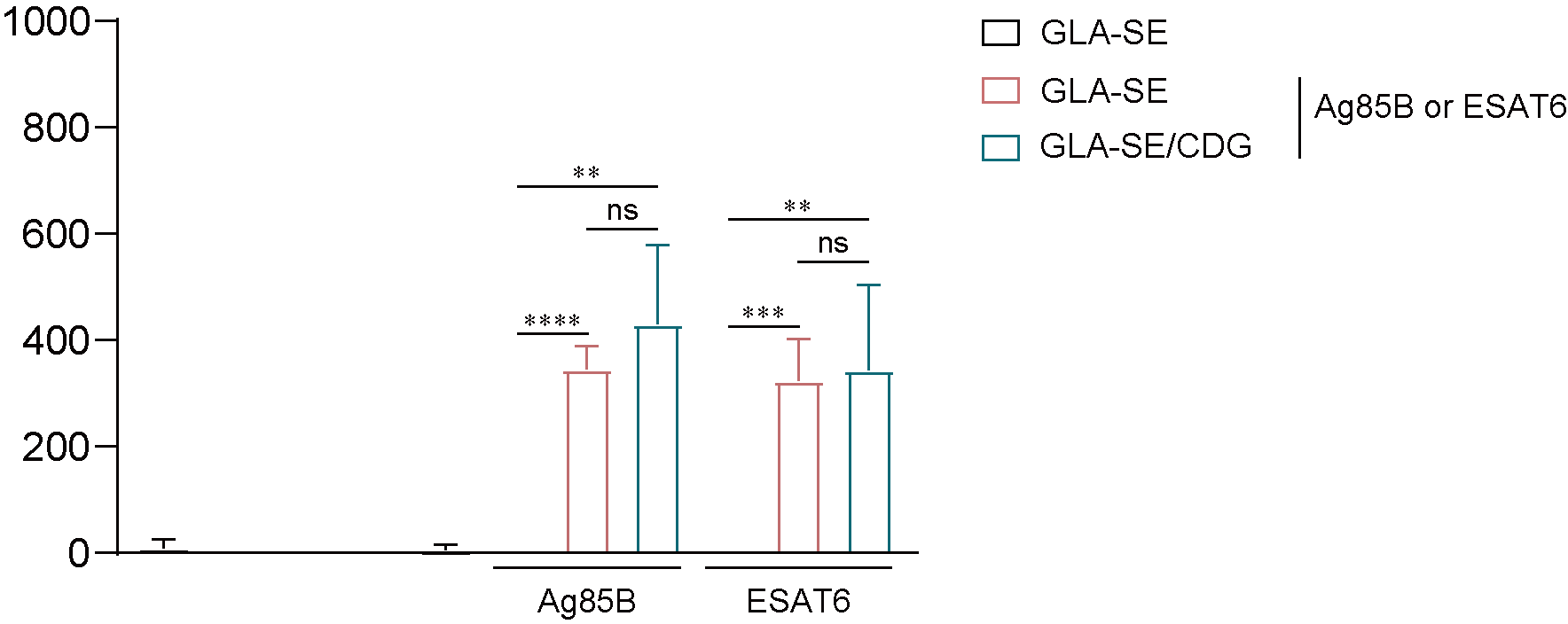


**Supplementary Figure 11. IL-17A production in the lung cells of mice immunized with Ag adjuvanted in GLA-SE/CDG upon Ag stimulation.** Levels of IL-17A secreted by lung cells in all treatment groups in response to ESAT6 (1 μg/ml) or Ag85B (1 μg/ml) stimulation as detected by ELISA. All bar graphs show the means ± SD of 4 samples. The data are representative of a single experiment. n.s. not significant.


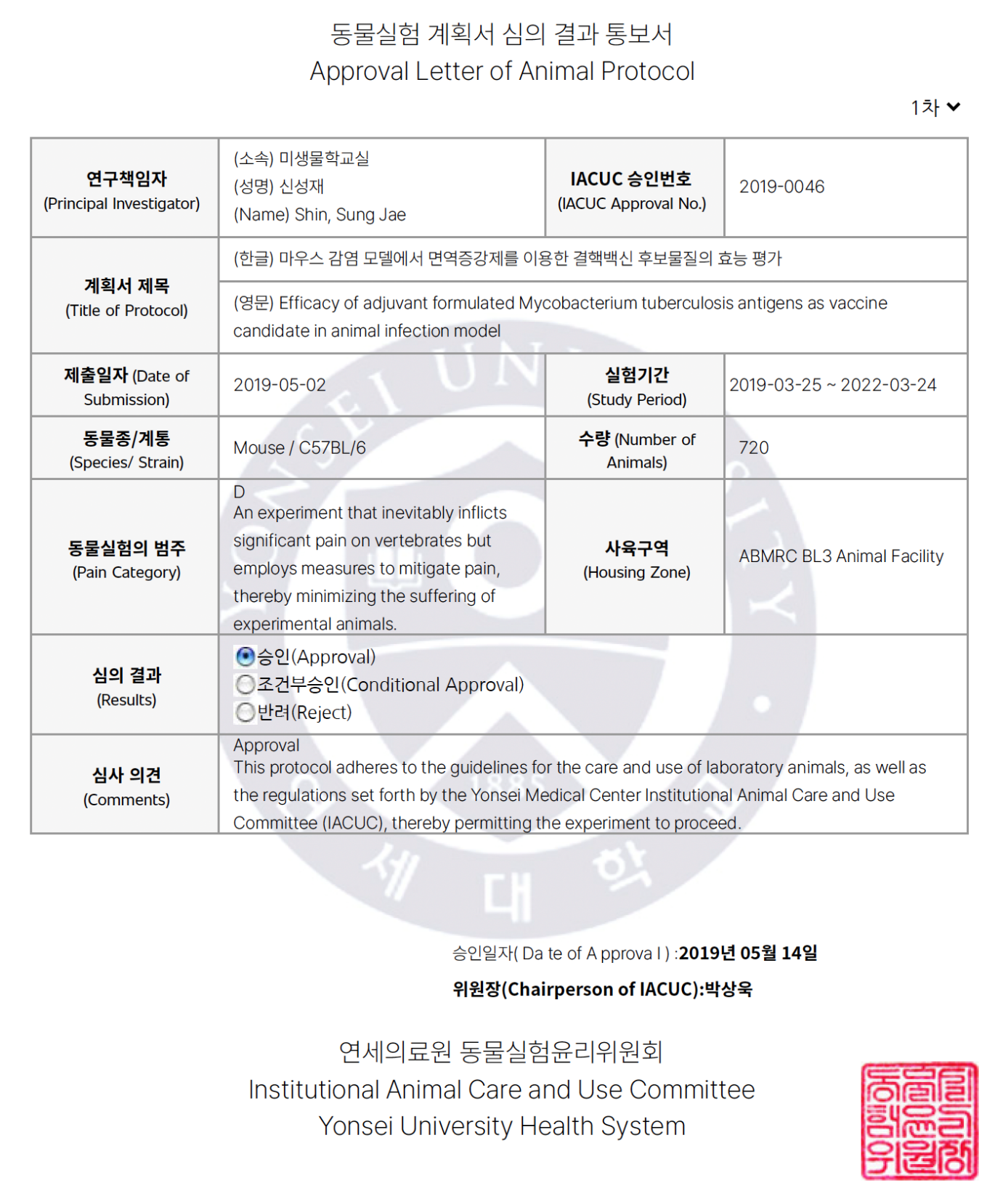


**Supplementary Figure 12. Approval certificate of animal protocol.**
